# An exopolysaccharide-producing novel *Agrobacterium* *pusense* strain JAS1 isolated from snake plant enhances plant growth and soil water retention

**DOI:** 10.1038/s41598-022-25225-y

**Published:** 2022-12-09

**Authors:** Jaspreet Kaur, Gaurav Mudgal, Kartar Chand, Gajendra B. Singh, Kahkashan Perveen, Najat A. Bukhari, Sandip Debnath, Thotegowdanapalya C. Mohan, Rajulu Charukesi, Gaurav Singh

**Affiliations:** 1 University Institute of Biotechnology, Chandigarh University, Gharuan, Mohali, Punjab 140413 India; 2 Department of Botany and Microbiology, College of Science, King Saud University, Riyadh, 11495 Saudi Arabia; 3 Department of Genetics and Plant Breeding, Palli Siksha Bhavana (Institute of Agriculture), Visva-Bharati University, Sriniketan, Birbhum, West Bengal 731236 India; 4 Department of Biotechnology and Bioinformatics, School of Life Sciences, JSS Academy of Higher Education and Research, Bannimantapa Road, Mysore, 570015 India; 5 Stress Signaling to the Nucleus, CNRS-Institute of Molecular Biology of Plants, 12 Rue du General-Zimmer, 67000 Strasbourg, France

**Keywords:** Biological techniques, Biotechnology, Microbiology, Molecular biology, Plant biotechnology

## Abstract

A peculiar bacterial growth was very often noticed in leaf-initiated tissue cultures of *Sansevieria* *trifasciata,* a succulent belonging to the Asparagaceae family. The isolate left trails of some highly viscous material on the walls of the suspension vessels or developed a thick overlay on semisolid media without adversities in plant growth. FTIR identified this substance to be an extracellular polysaccharide. Various morphological, biochemical tests, and molecular analyses using 16S rRNA, atpD, and recA genes characterized this isolate JAS1 as a novel strain of *Agrobacterium* *pusense.* Its mucoidal growth over Murashige and Skoog media yielded enormous exopolysaccharide (7252 mg l^−1^), while in nutrient agar it only developed fast-growing swarms. As a qualifying plant growth-promoting bacteria, it produces significant indole-3-acetic acid (86.95 mg l^−1^), gibberellic acid (172.98 mg l^−1^), ammonia (42.66 µmol ml^−1^). Besides, it produces siderophores, *1-aminocyclopropane-1-carboxylic* *acid* *deaminase*, fixes nitrogen, forms biofilms, and productively solubilizes soil inorganic phosphates, and zinc. Under various treatments with JAS1, wheat and chickpea resulted in significantly enhanced shoot and root growth parameters. PGP effects of JAS1 positively enhanced plants’ physiological growth parameters reflecting significant increments in overall chlorophyll, carotenoids, proline, phenols, flavonoids, and sugar contents. In addition, the isolated strain maintained both plant and soil health under an intermittent soil drying regime, probably by both its PGP and EPS production attributes, respectively.

## Introduction

Endophytes are organisms that reside in and variously colonize their plant hosts. Microbial endophytes are bacterial and fungal entities that reside within plant tissues establishing symbiotic and non-symbiotic relationships with the host, and generally do not impose any negative influence on the host’s growth^1–4^. Although newer studies have included pathogens following endophytic lifestyles^5,6^, many others offer a plethora of growth benefits to the host, more importantly in the bio-availability of mineral nutrients and protection from abiotic and biotic stressors^7^. Many endophytes also offer key bioresources to agriculturally, medicinally, and industrially relevant products and processes^8–18^. Rhizobacteria include various groups of soil-borne bacteria that positively and/or negatively impact plant growth. Plant growth-promoting rhizobacteria (PGPR) establish rhizospheric and/or endophytic associations with plants exhibited well in some with the formation of root nodules in legumes where they assist the plants in fixing atmospheric nitrogen into ammonia. Root nodulation, however, is not a necessity as many such bacteria colonize within the leaf, stem, root, and even seeds of many legumes and non-legumes^19,20^. PGP properties of endophytes are understood in their ability to solubilize soil minerals, *1-aminocyclopropane-1-carboxylate* *(ACC)* *deaminase* activity, siderophore production, auxin synthesis, etc.^21,22^. Some microbes are known to fabricate and release, besides other bioactives, certain low to high molecular weight carbohydrate biopolymers called extracellular polysaccharides (EPS). In plant-associated bacteria, EPS secretions enhance (i) their interaction/adherence, movement, and colocalization within the plant systems by forming biofilms; (ii) water retention capacity of soil and host root and (iii) as well in availing other many benefits viz., protecting host(s) from pathogens, substratum/nutrient signaling, and possibly as well plant acclimatization to environmental cues and so on^21,23^. Many bacterial EPS secretions have seen translations into a range of commercial food, agriculture, biomedical and cosmetic applications with an increasing market value^24–28^.

Agrobacteria include both plant growth-promoting species and plant pathogens as well, many of which have variously helped plant biotechnologists and molecular biologists. For more than a century, the genus *Agrobacterium* had witnessed several taxonomic reshufflings, and with currently 14 members, it majors in eight plant pathogens, two nonpathogens, and four that remain yet uncharacterized^29^. The species *Agrobacterium* *pusense* (AP), first isolated from chickpea rhizosphere (NRCPB10^T^)^30^, initially sat within the genus *Rhizobium* but was later shifted to *Agrobacterium* based on housekeeping DNA barcodes^31,32^. Newer AP strains have increasingly been reported recently, of which only a few were studied for their PGP assets^33–44^.

*Sansevieria* as a genus (maintained in the manuscript hereafter; although very recently sunken into genus *Dracaena*^45^), comprises slow-growing succulent plants, commonly referred to as ‘snake plants’ within the family Asparagaceae and include around 70 species of the principally considered ornamentals native to South Africa, South Asia, and Madagascar. They are shade tolerant, can withstand arid, semi-arid, high salt soils, and grow mostly through stolons^46^. The genus wholesomely amazes plant scientists with the ability to run photosynthesis following the Crassulacean Acid Metabolism (CAM) which prolongs its oxygen release even in dark, and in being resilient to longer soil dryness, pathogenesis and herbivory as well, all of which also indeed qualify them as perfect indoor and garden ornamentals^47,48^. *Sansevieria* fibers have been traditionally used in making ropes, reinforcing material composites, and as fillers, especially for the traditional Nigerian rubber^49–51^. They have been used in suture material for closing intraoral incisions, a practice mentioned variously in the age-old Indian Ayurvedic compendium, the ‘Sushruta Samhita’^52,53^. Ethnopharmacology supports the use of *Sansevieria* preparations in various ailments, such as earache, menstrual and labor pains, malaria, and conditions related to the heart, liver, kidney, lung, skin, gastroenteric tract^54–62^ and also more profoundly as a remedy to snake bites^63–66^. *Sansevieria* *trifasciata* (ST) has been appraised by NASA for its air pollution-absorption potentials^47,67^, and other many species in the genus also variously show bioremediation prospects^68–72^. In addition, numerous phytochemicals and potent bioactives have been identified in many *Sansevieria* species^73–75^.

Many succulent plants have emerged as hotspots of research primarily focused on their phenomenal resilience to various environmental cues^76–81^. Other investigational leads explore interactions with microbionts which variously augment resilience attributes in these plants^82–85^. Research in the genus Sansevieria however witnesses a slow pace in current developments majoring only with its phytochemical^73–75^ and fiber reserves^86^. Recent literature depicts a recessed trend of investigations especially those warranted on various stressors and mechanisms that allow survivability of Sansevieria plants. Perhaps, studies on microbionts, their interaction, and significance in these plants also remained largely overlooked. The only work to date refers to the isolation of two PGP *Bacillus* *cereus* strains from *Sansevieria* *kirkii* based on their trimethylamine removal efficiency^87^. We attempt to fill the lacuna with relevant studies on various succulent plants at our end.

Previously, we reported that relatively higher daytime temperatures effectuate quick whole plant regeneration from in vitro rooted ST leaf explants, and this is remarkably without the exogenous supplementation of any cytokinins^88^. Other than this, we reckon that many of our tissue culture trials with ST routinely witnessed contamination from a peculiar bacterial growth, forming some highly viscous material on the agar and liquid suspension media. The bacteria however imposed no detriments on the productive regeneration of plants. Our curiosity-driven studies explore various prospects associated with this bacterial isolate, especially in light of the research gaps in Sansevieria outlined before. At the outset, this paper presents the isolation and identification of this bacterium as a novel *Agrobacterium* *pusense* endophyte strain, JAS1 from the leaves of *Sansevieria* *trifasciata* var. *Laurentii*. We characterized its in vitro growth patterns, EPS production potential, and PGP traits and also variously document its prospective application in improving both crop and soil health.

## Materials and methods

### ST leaf tissue culture

*Sansevieria* *trifasciata* (var. *Laurentii*) (= ST) leaves were procured from a rhizome cluster at the botanical garden in Chandigarh University, Mohali (Punjab). All local, national, international guidelines and legislation were adhered while the collection and culturing of ST plants in this study. Surface sterilization and in vitro establishment of ST was followed from our previously reported protocol^88^. Briefly, one fully expanded healthy green leaf was excised from each plant, cut into 5–6 cm segments, and washed thoroughly under running tap water (30 min), followed by a vigorous rinsing in a mild detergent solution (2–4 drops of Tween-20 in 100 ml tap water) and later clearing with 30 min wash under running tap water. Under the laminar flow hood, the leaf segments were rinsed in Dettol (aq. 1% v/v, 1 min) and cleared with sterile distilled water (SDW) (2 × 5 min washes) followed by sequential treatments with mercuric chloride (0.1% v/v), 70% ethanol (each for 45 s) and a final wash with SDW (3 × 5 min). Trimmed leaf explants (1 cm^2^) were aseptically transferred to semisolid or liquid (with/without 0.8% gelling agent res.) Murashige and Skoog (MS) media supplemented with sucrose (3%) and various growth regulators. The culture room was set to standard plant tissue culture (PTC) conditions (22 ± 2 °C temperature, 60 to 65% rel. humidity, and 16:8 h’ light/dark photoperiods).

### Endophyte isolation

Leaf-initiated ST tissue cultures seen with contamination within 3–4 weeks post-inoculation were normally rejected for endophyte isolation. Long-term (≥ 4 weeks) cultures when exhibiting either a peculiar bacterial ooze (from the sides of the explant and later spreading to the media surface) on semi-solid MS and/or a highly viscous gel-like biomass in liquid MS were selected. A loop-full culture from the selected vessels was streaked over NA (nutrient agar), and MSA (MS-agar) and incubated for 2 days (under PTC conditions). Alternatively, a standard sampling method^89^ was also used to procure similarly morphed (oozing) colonies from healthy, surface sterile ST leaf segments crushed in ice-cold 1× PBS and inoculating aliquots from the serial dilutions on NA and/or MSA. Single colonies were respectively inoculated over NB (nutrient broth) and MSB (MS broth) for various other methods as well as to facilitate cryo-storage in glycerol (15% in respective MSB and NB).

### Biochemical characterization

Biochemical assays such as tests for catalase, methyl red, indole, citrate utilization, Voges–Proskauer, starch hydrolysis, urease, oxidase, nitrate reduction, motility, H_2_S production, tween 20 and tween 80 hydrolysis, α-ketolactoseThe qualitative utilization, carbohydrate utilization and growth on NaCl were performed with standard protocols described in the manual by Cappuccino and Sherman^90^. All tests were performed thrice.

### Enzyme activity assays

All enzyme activity screening assays employed standard methods. For screening cellulolytic (= cellulose degradation) activity, carboxymethylcellulose (CMC) agar plates were spot inoculated with the bacterial isolate and incubated for 24 h (at 28 °C) following which media was overlaid with iodine solution. A clear halo zone infers positive cellulolytic activity^91^. For proteolytic activity screening, the bacterial isolate was spot inoculated on plates with skim milk agar media (Himedia, Mumbai, India) which if develops a clear halo zone around its colony(s) (upon incubation at 28 °C for 24 h) would indicate positive proteolytic activity^92^. Lipase activity was assayed by spot inoculating the isolate on TBA (Tributyrin agar) base supplemented with 1% Tributyrin (Himedia, Mumbai, India). A positive lipase activity would be inferred from a clear zone around the bacterial colony(s) following an incubation regime of 24–48 h (at 28 °C). Pectinase assay employed inoculating the bacterial isolate over Pectinase Screening Agar Medium (PSAM). Plates were incubated at 28 °C for 2 to 3 days and later flushed with 3–4 ml of iodine solution. A halo zone around the bacterial growth indicates positive pectinase activity^93^. Amylase activity was screened on starch agar medium (SAM) spot inoculated with the bacterial isolate (and incubated at 28 °C for 1 to 2 days). Plates were flooded with 1% iodine solution for 20 min. A clear yellow zone around the growth indicates positive amylase activity^94^. Other details on media composition are provided elsewhere (see media and reagents in supplementary file).

### Bacterial motility tests

The motility test was performed using two standard methods: the semi-solid agar and the wet mount method^95^. The former used a fine loop to stab bacteria vertically deep into an agar butt (SIM Medium Butt; Himedia, Mumbai, India) which was incubated overnight (at 28 °C) following the manufacturer’s recommendations. The other method used a wet mount of bacteria over a glass slide and viewed for growth patterns under a light microscope (Metzer, Vision plus-5000 DPCT). Both methods used fresh inoculums raised from a single colony on NB media.

### Antibiotic sensitivity tests

Antibiotic sensitivity assays for the bacterial isolate used a standard disc diffusion test^96^ following the CLSI guidelines^97^. Bacterial starter culture raised overnight from a single colony (in NB, 28 °C, 200 rpm) was spread plated on Muller Hinton agar (Himedia, Mumbai, India). After about 30 min of inoculum soaking, susceptibility discs for various antibiotics (Himedia) were placed on plates and incubated overnight in dark at 28 °C. The observed zone of inhibition was measured as diameters (= extent of bacterial susceptibility to the antibiotic) otherwise depicting antibiotic resistance. Results were expressed as means of the means from three replicates each arriving from three trials per antibiotic assayed. *A.* *rhizogenes* strain A532 was used for reference. Antibiotic sensitivity data of the isolate was compared with that reported for AP strain NRCPB10^T^ and MB17-a^30,33^.

### Molecular identification of the isolate

Pure culture of the bacterial isolate JAS1 was sent to MTCC, CSIR-IMTECH, Chandigarh for 16S rRNA sequence-based identification. For further validation, JAS1 genomic DNA was used as a template for PCR amplification of two housekeeping genes, *recA* (with primers recAF: 5′-ATGGCACAAAATTCTTTGCGTCTCGTAGAG and recAR: TCAVCCTTCGTCACCRTCGCCGTCATCGC) and *atpD* (with primers atpDF: ATGGCTAAGGCAGCTACCCCMAAGAAAACC and atpDR: TCAGGCAGCYTCGGCAGCCAGCTTCTTSGC). PCR reactions consisted of 200 mM PCR buffer, 1.5 mM MgCl_2_, 10 pmol µl^−1^ each primer (Bioserve Biotechnologies India Pvt. Ltd., Hyderabad, India), 10 mM dNTPs, 5 units of *Taq-*DNA polymerase (Himedia, Mumbai, India) and molecular biology grade water (Himedia, Mumbai, India) up to 50 µl total volume per reaction. PCR program (run on BIORAD S1000™ thermal cycler) included steps with initial denaturation (5 min, 95 °C) followed by 32 cycles each with intermittent denaturation (1 min, 95 °C), annealing (1.5 min, 60 °C) and extension (1 min, 72 °C); altogether followed with a final extension step (10 min, 72 °C). Amplicons were gel extracted (Himedia, Mumbai, India) and ligated to pGEM-T easy vector (Promega, New Delhi, India) which was then transformed to DH5-α competent bacteria following the heat-shock protocol. Recombinant clones were screened using blue-white screening and validated for inserts using colony PCR and/or restriction enzyme digestions of the plasmids. Plasmid isolates with respective gene inserts were sequenced using universal M13 primers (Eurofins Genomics India Pvt., Bangalore). Sequences were screened and trimmed for vector backbone sequences using the VecScreen tool (https://www.ncbi.nlm.nih.gov/tools/vecscreen/) and then analyzed for chromatograms using the CHROMAS software (version 2.6.6, Technelysium Pty Ltd). BLAST for sequence similarity search was carried out with GenBank (https://www.ncbi.nlm.nih.gov/genbank/) and EzBioCloud databases (https://www.ezbiocloud.net/), respectively. Clustal W program was used to align highly similar sequences and MEGA (version 11.0.11, https://www.megasoftware.net/downloads/dload_win_gui) ^98^ for constructing phylogenetic trees based on various algorithms. All outlined kit-based procedures follow the manufacturer’s recommendations.

### EPS production and purification

Crude EPS was isolated from the oozing spread of JAS1 that developed over 10 days on MSA plates (inoculated with a revived single colony on NA, 28 °C). Drooping volume (~ 10 ml directly from the lid of the inverted plate) was collected into a falcon tube. To ease bacterial separation, crude EPS was double diluted with SDW. Near clear supernatant was resolved after centrifugation (10,000 rpm, 30 min, 4 °C on REMI-CPR-24 PLUS, Remi, Mumbai, India), and further clarified through 0.45 µm filter discs on an ultra-filtration assembly (Tarsons, Kolkata, India). EPS was precipitated with two volumes of ice-cold 100% absolute ethanol and overnight stored at −20 °C. Later, EPS was spool-collected into a fresh falcon tube and centrifuged (10,000 rpm, 30 min, 4 °C). Pellet was left for drying at 60 °C and later mixed in 5–10 ml SDW and dialyzed through cellulose membrane (10–14 KDa MWCO; Himedia, Mumbai, India) for 48 h at constant stirring in 2 l ultra-filtered SDW (at 22 °C). EPS was recovered and lyophilized in a freeze dryer (Allied-Frost, New Delhi, India) to result in a powdered preparation. This was weighed to account for the total EPS recovered from 10 ml of droop collected from the MSA plate. The resulting EPS was also estimated using a standard acid hydrolysis test^99^. Other than this small-scale EPS production regime used inoculating 50 µl JAS1 starter (overnight raised on NB using a NA raised single colony; 28 °C) in 50 ml MSB in several 250 ml vol. flasks for a month’s incubation (28 °C, 120 rpm). At definite intervals, flasks were removed from incubations and instantly processed for EPS recovery and purification using the same steps outlined before with proportionate volumetric adjustments and stringencies to avoid any volume loss. Freeze-dried weights were measured in respective purifications to account for final EPS yield (w/v) at various day intervals post-inoculation. All tests were repeated thrice and the results were shown as means of the means. These EPS preparations were further used in FTIR analysis and other essays when required. Based on the recovery from small-scale EPS production trials (above), a liter’s scale batch production was attempted in MSB inoculated with 1 ml overnight starter culture of JAS1 (raised from a single colony over NA) in a 5-l Erlenmeyer flask and incubated for 10 days in an orbital shaker (120 rpm, 28 °C on REMI CIS-18plus, Kolkata, India). The whole (highly viscous) culture was doubly diluted with ultra-filtered SDW and centrifuged thrice (10,000 rpm, 30 min, 4 °C) to avail a near clear supernatant (minimizing any possible loss in volume while transfer steps). EPS was precipitated by mixing 2 volumes of ice-cold 100% absolute ethanol followed by overnight incubation at −20 °C. Precipitated EPS was spool-transferred to fresh pre-weighed falcon tubes (50 ml each). Following centrifugation (10,000 rpm, 1 h, 4 °C) the resultant EPS pellet was dried completely in an orbital shaking incubator (45 °C, 150 rpm. 3 days). Total dry weight was measured to account for the w/v EPS yield.

### FTIR analysis of EPS

Purified and lyophilized EPS sample (2 mg) was mixed with 200 mg dry KBr, pressed into a 16 mm diameter mold^100^, and followed with chemical composition analysis by Fourier Transformed InfraRed (FTIR) spectroscopy (on a Spectrum Two FT-IR Spectrometer from PerkinElmer, Mumbai, India) at the Department of Chemistry, Chandigarh University. FTIR spectrum was recorded in the region 4000–400 cm^−1^ at a resolution of 1 cm^−1^.

### XRD of EPS

Lyophilized EPS powder was studied for ascertaining its amorphous and/or crystalline nature using X-ray diffractometry^101^ performed at the UCRD, CU. The diffractogram (Bruker D8 Advance, Mumbai, India) was examined with Ni-filtered Cu Kα radiation scan ranges of two-theta angles (5°–80°) at RT, 40 kV voltage, and 25 mA current. The crystallinity index (CI_xrd_) was ascertained using the following standard formula^102^:

$$CIxrd=\frac{\sum \mathrm{Acrystal}}{\sum \mathrm{Acrystal}+\sum \mathrm{Aamorphous}}$$

### Microscopy

Light microscopy was variously performed using a phase-contrast microscope (Vision plus-5000 DPCT, Metzer, Mumbai, India) for magnified observations of the bacterial isolate to account for its morphological and motility features. Stereomicroscope (Nikon-745T fitted with a 5 Megapixel digital camera, Nikon, Mumbai, India) sufficed in recording stills of plant roots. Surface characteristics of the JAS1 isolate and its produced EPS were analyzed at the Scanning Electron Microscopy (SEM) facility of the University Center for Research and Development (UCRD), Chandigarh University using a standard method^103^. In brief, for JAS1, a 1 ml aliquot from the overnight grown culture on LB broth was centrifuged at 4500 rpm for 5 min on a desktop centrifuge (RM-03 plus, REMI, Mumbai, India). For prefixation, the bacterial pellet was washed thrice in 1× PBS (10 mM; pH 7.2), resuspended in 0.25% glutaraldehyde (in PBS 10 mM, pH 7.2), and incubated for 30 min. Pellet was washed 4 times with SDW and treated with osmium tetroxide (0.1% w/v) for 30 min at RT. Pellet was rewashed thrice with PBS and dehydrated through a series of increasing ethanol concentrations (30–100%; 10 min each). A 10 μl aliquot was processed for imaging. For EPS, a 3–4 mg sample of lyophilized EPS was applied onto a carbon-coated stub and gold-sputtered. Images were recorded at an accelerating voltage of 10 kV on a JEOL TouchScope series (model JSM-IT500, JEOL, New Delhi, India).

### EPS solubility test

To ascertain solubility in different solvents, 5–30 mg of freeze-dried pure EPS was individually mixed with 2 ml of various solvents viz., water, alcohol, acetone, ethyl acetate, methanol, DMSO, chloroform, hexane, butanol, ethanediol, and benzene. The mixture was left on a rotatory mixer for 2 days, centrifuged (10,000 rpm, 15 min, 4 °C), and evaluated on the visual account.

### Plant pathogenicity test

Carrot roots were availed fresh from a village (Gharuan) farm in the close vicinity of Chandigarh University and were followed with standard surface sterilization and bacterization protocol^104^. In brief, surface sterile 1 cm thin, cross-sectioned root discs were established on NA overlaid with a spread of JAS1 (1 × 10^4^ CFU ml^−1^) and incubated in a PTC room with standard conditions mentioned before. Root discs were observed for 2 weeks for any pathological symptoms. All trials were performed thrice each with three replicates per trial.

### PGP characterizations

#### Phosphate solubilization

JAS1 isolate was tested for phosphate solubilization using a standard Pikovskaya’s agar plate protocol^105^ with slight modifications. JAS1 isolate was spot inoculated on Pikovskaya’s agar (Himedia, Mumbai, India) supplemented with tricalcium phosphate as an insoluble P source. The culture was incubated at 28 °C for up to 10 days for the development of a clear halo around the colonies which infer positive phosphate solubilization measurable as an index using the following formula

$$\text{Phosphate}\, \text{solubilization} \,\text{index }(\text{cm})=\frac{\text{Colony} \,\text{diameter} \left(\text{cm}\right)+\text{Halozone} \,\text{diameter}\,(\text{cm})}{\text{Colony} \,\text{diameter }(\text{cm})}$$

Qualitative analysis of soluble phosphate was followed using standard methods^105–107^ with some modifications. In brief, JAS1 isolate was cultured in 250 ml flasks with 50 ml of National Botanical Research Institute’s Phosphate (NBRIP) medium (supplementary file). The culture was incubated in an orbital shaker (28 °C, 180 rpm) for 10 days followed with an intermittent assay for soluble phosphates released in the NBRIP medium. Briefly, 2 ml culture aliquots were withdrawn at 48, 120, 160, and 216 hourly intervals and centrifuged (10,000 rpm, 10 min). Cell-free supernatant (1 ml) was then mixed well with 2 ml of 1% boric acid and 3 ml of freshly prepared molybdate reagent (supplementary file) and incubated undisturbed (RT, 40 min). Samples were read for OD_700_ values spectrophotometer (UV-1800, Shimadzu, New Delhi, India). Quantification of available phosphates followed with plotting OD_700_ values on a standard curve (potassium dihydrogen phosphate in the range of 2–30 µg ml^−1^). All tests were repeated thrice with four replicates per test.

#### *ACC deaminase* production

Production of *ACC* *deaminase* by JAS1 isolate was detected with its growth on DF minimal salt medium (supplementary file) supplemented with 2 g l^−1^ (NH_4_)_2_SO_4_ and incubated for 72 h at 28 °C^108^.

#### Siderophore production

Screening and evaluation of siderophore production used freshly prepared Chrome Azurol S (CAS) reagent. As per the norm, any trace iron in all culture and test vessels were removed by rinsing them overnight in 3 mol l^−1^ HCl and washing later with SDW^109^. Qualitative test for siderophore production followed the standard CAS agar plate method^110^. Briefly, nutrient agar (NA) plates supplemented with 10% CAS reagent were streaked with the bacterial isolate and incubated at 28 °C for a week. Formation of a yellow to orange halo around the streak infers a positive result. Quantitation of siderophore production used a modified microtiter plate method^111^. Hundred microliter aliquots were withdrawn from an NB grown culture of the isolate at various time intervals (48, 120, 160, and 216 h), centrifuged (at 10,000 rpm, 10 min), and respectively mixed with 100 µl freshly prepared CAS reagent. After 20 min of incubation, formation of a yellow to orange colored complex in test samples infers a positive siderophore production. The OD_630_ values of the test samples were recorded on a microplate reader (RT2100C, Rayto, Guangming New District, China). The experiment was performed thrice with four replicates per test. Siderophore production was quantified using the standard formula^112^:

$$\mathrm{Percent Siderophore Unit }\left(\% \mathrm{SU}\right)= \frac{({\mathrm{A}}_{\mathrm{r}}-{\mathrm{A}}_{\mathrm{s}})}{({\mathrm{A}}_{\mathrm{r}})}\times 100$$

Here: A_r_ is the absorbance of reference (uninoculated broth and CAS reagent); A_s_ is the absorbance of sample (inoculated broth and CAS reagent).

#### Indole-3-acetic acid (IAA) production

IAA production was assayed using a standard method^113^ with slight modifications. Bacterial isolate was cultured in NB supplemented with 0.1% tryptophan and incubated for 10 days (at 28 °C, 120 rpm). Culture aliquots were withdrawn at different time intervals (48, 120, 160, and 216th h) and centrifuged to remove the pellet (10,000 rpm, 15 min). The supernatant (1 ml) was properly mixed with 2 ml of freshly prepared Salkowski reagent (1 ml FeCl_3_ and 50 ml 35% HClO_4_)^114^ and allowed to stand in dark for 30 min. Pink coloration in the test samples infers IAA production which was quantified spectrophotometrically (OD_530_) using a standard curve for commercially purchased IAA (Sigma-Aldrich, USA). All tests were repeated thrice with four replicates per test.

#### Ammonia production

Ammonia production was studied using a standard protocol^90^. Bacterial isolate was inoculated in peptone water (Himedia, Mumbai, India) and incubated for 10 days (28 °C, 120 rpm). Test aliquots were withdrawn at different time intervals (48, 120, 160, and 216th h) and centrifuged to remove the pellet (10,000 rpm, 15 min), while 1 ml of the supernatant was mixed with 50 µL Nessler’s reagent (Thermofisher Scientific, New Delhi, India). Yellow to brown coloration in test aliquots depicts positive ammonium production which was quantified spectrophotometrically (OD_450_) using a standard curve for commercially purchased ammonium sulfate (Himedia, Mumbai, India). All tests were repeated thrice with four replicates per test.

#### Gibberellic acid (GA) production

GA production by JAS1 was elucidated by employing a standard spectrophotometric assay^115^. In brief, 2 ml supernatants from culture (at 28 °C, 120 rpm) aliquots were withdrawn at various time intervals (48, 120, 160, and 216 h) after centrifugation (10,000 rpm, 10 min) and mixed with 280 µl of 1 M zinc acetate and later with 280 µl of 10.6% of potassium ferrocyanide solution under vigorous vortexing. Centrifugation (4500 rpm, 10 min) led supernatant was equally mixed (v/v) with 30% HCl (added slowly) and incubated (at RT for 75 min). Absorbance was recorded in a spectrophotometer (OD_254_) against a blank containing 5% HCl and was fitted to a standard curve for commercial purchased GA (20–200 μg ml^−1^, Himedia, Mumbai, India).

#### Zinc solubilization

For the preliminary inference on the zinc solubilization activity of JAS1, a standard plate assay was employed^116^ that requires spot inoculation of the isolate over a semisolid basal media (supplementary file) mixed with 0.1% zinc oxide (insoluble, Himedia, Mumbai, India) and incubation at 28 °C for a week to observe any clear zones around the spot colony inferring zinc solubilization which can be measured as below

$$\mathrm{Zinc solubilization index }(\mathrm{cm})=\frac{\mathrm{Colony diameter }\left(\mathrm{cm}\right)+\mathrm{Halozone diameter }(\mathrm{cm})}{\mathrm{Colony diameter }(\mathrm{cm})}$$

Alternatively, quantification of solubilized zinc in JAS1 inoculated broth (media as above) was performed using a standard Atomic Absorption Spectrometry (AAS) method^116^. Samples were withdrawn at different time intervals (48, 120, 160, and 216th h) and supernatants (10,000 rpm, 15 min) were fed to a Perkin Elmer AAS (Jeeva Labs Pvt. Ltd., Nalagarh, Himachal Pradesh).

#### Potassium solubilization

Potassium solubilization of the bacterial isolate was studied on Aleksandrow agar media (supplementary file) by a standard spot plate assay^117^ inoculated with a loop-full overnight grown culture and incubated at 28 °C. Clear zones around the colonies confirm positive potassium solubilization. The diameter of clear zones deduced the Khandeparkar’s selection ratio as below:

$$\mathrm{Khandeparkar}{\text '} {\rm s selection ratio} =\frac{\mathrm{Diameter of zone of clearance }(\mathrm{D})}{\mathrm{Diameter of growth }(\mathrm{d})}$$

#### Nitrogen fixation

The nitrogen-fixing ability of the bacterial isolate was verified using a standard method based on the ability of bacteria to grow on nitrogen-free media^118^. In brief, bacterial isolate was streaked on Jensen media (supplementary file) and incubated (at 28 °C) for a week. The growth of bacteria with glistening colonies and/or streaks on the above media infers positive nitrogen fixation. The test was repeated thrice.

#### Hydrogen cyanide production

Production of hydrogen cyanide by bacteria was ascertained using a standard method^119^. Bacterial isolate was streaked over NA plates supplemented with glycine (4.4 g l^−1^) and was fixed under a suitably sized, autoclaved Whatman filter paper disc (No. 1) freshly soaked in 2% sodium carbonate solution (prepared in 0.5% picric acid). The plates were incubated in dark (at 28 °C for 4–5 days), and when developing a dark orange to red coloration on filter paper, inferred positive HCN production by the isolate. The assay was repeated thrice.

#### Biofilm formation

Congo red agar (CRA) method is a qualitative method for screening biofilm formation (BF) ability in microorganisms. Post streak inoculation of the bacterial isolate, CRA plates (supplementary file) were incubated at 28 °C for 24 h. Crystalline blackening of colonies with dry consistency would vouch for the positive bio-film forming by bacteria^120^.

### In vitro effects of JAS1 on ST leaf explants

ST leaf explants were established in jam jars following our standardized PTC protocol^88^. Post root formation (at 3 weeks after ST explant establishment), a test set of explants were primed with JAS1 (1 × 10^4^ CFU ml^−1^ raised in NB) while those in control were treated with sterile NB. Cultures were maintained under PTC room conditioning discussed before. Observations were recorded post 5 weeks of JAS1 treatments. The experiment was repeated thrice with five replicates each within the test and control sets.

### Effects of JAS1 priming on ex vitro wheat growth

PGP effects of the isolate were validated experimentally in a controlled glasshouse setting (25–27 °C, 70–90% relative humidity and under natural photoperiods) following a completely randomized design (CRD). Seeds (10 g) of a commercial wheat variety (UNNAT PBW 343) were purchased locally (Grain market, Mohali, Punjab, India) and soaked overnight in 100 ml of ultra-filtered (0.22 μm pore sized, nylon filter paper discs, Millipore, Bengaluru, India) tap water (TW). Seeds were divided into test and control sets (100 seeds each). The test set was primed with 100 ml of TW resuspended JAS1 culture (1 × 10^8^ CFU ml^−1^; raised overnight from a single colony on NB; 28 °C, 120 rpm, dark). The control set (uninoculated with JAS1) was treated with just the same volume of TW. The seeds (70 numbers per set) were then sown directly (1.0 cm deep) into a soil bedding (prepared with mesh sorted and autoclaved garden soil) layered (soil depth 4 cm) onto plastic trays (43 × 34 × 7 cm). Soil beds were spray watered on the same day post sowing with ca. 150 ml TW, thereafter every day with 50 ml (TW). This included as well booster dosing test soil beds with spray inoculation of JAS1 (50 ml TW with 1 × 10^8^ CFU ml^−1^) once per week for only initial three weeks while control beds were treated only with 50 ml TW. Seedling growth was monitored and recorded periodically. After 8 weeks, various growth paraments viz., overall fresh and dry weight (FW and DW respectively), and primordial length were compared following soil drain-out. In another similarly designed trial, post 4 weeks’ treatments as in above, soil beds were left unwatered for a week to observe seedling performance under a soil drying regime. Each of the above trials was repeated thrice.

### Effects of JAS1 on in vitro wheat growth

In vitro trials were designed each comprising a test set with three replicates of sterile PTC jam jars each with two surface-sterilized wheat seeds (var. UNNAT PBW-343) established over 50 ml semisolid MSA media carrying 1 ml fresh overlay spread of SDW resuspended bacterial pellet (1 × 10^4^ CFU) derived from an overnight grown starter culture of JAS1. In the control set, the JAS1 spread was replaced with SDW. Culture incubations were done for a month in the PTC room followed by finally recording growth parameters viz., shoot and root length, root branching, and root hair density and length. Standard PTC room conditions were maintained as mentioned earlier. Trials were run thrice following the above experimental regime.

### Effect of JAS1 on chickpea

Like in wheat, we also attempted to characterize the PGP effects of JAS1 on a legume crop. For this, seeds of a locally available commercial variety L550 of chickpea (*Cicer* *arietinum* L) were in transparent plastic pots (1 l) containing ca. 0.7 kg of finely sieved and autoclaved garden soil (as detailed before with wheat tray trial). About 25 g seeds were cleaned for 30 min with two drops of Tween-20 under running tap water followed by three rinses with ample TW. For the JAS1 test sets, seed priming involved imbibing and co-cultivating them together in 100 ml of TW resuspended JAS1 inoculum (1 × 10^8^ CFU ml^−1^, overnight, 28 °C) raised using an overnight starter culture grown from a single colony (in NB, 28 °C, 120 rpm, dark). Control sets were similarly imbibed in an equal volume of TW (devoid of JAS1). Each of the control and JAS1 treatment sets consisted of five pots (replicates) with three seeds sown per pot. On the 15th day post sowing, a booster dose of JAS1 (10 ml of TW resuspended 1 × 10^8^ CFU ml^−1^) was applied to each pot (except the control sets which were irrigated with 10 ml sterile TW) following a soil drench method^34^. All plants were irrigated with 30 ml of TW every third day till the 27th day (3 days before the drain out for final observations of roots). The experiment was repeated thrice with each trial scheduled to run for a month to record the effects on the shoot and root development.

### Soil water retention

Independent trials were attempted using 150 g of autoclaved and oven-dried garden soil-beddings prepared on plastic plates and drenched with 30 ml of either (i) MSB resuspended overnight grown JAS1 inoculum (1 × 10^8^ CFU ml^−1^), or (ii) EPS preparation derived by SDW resuspending the dried powdered ethanol precipitate from the centrifuged supernatant of a 10-day MSB grown JAS1 culture (= EPS treatment), or (iii) SDW (= control treatment). The respective plates were left in a laminar airflow hood at 28 °C and the blower speed was set to 0.45 ms^−1^. Water retention percentage was derived by periodically weighing the plates and evaluating water retained after a periodic water loss in each replicate put under the three different treatments. All experimental trials were repeated thrice with three replicates per treatment.

### Biochemical estimates of plant growth

Tray and pot trials with and without JAS1 priming treatments respectively carried out for wheat and chickpea (see above) were randomly sampled for standard biochemical assays as under.

#### Total proline content

As per a standard method (referred in^121^), 100 mg of shoot biomass was thoroughly crushed and mixed with 5 ml of 3% sulfosalicylic acid. From the supernatant retrieved after centrifugation (at 10,000 rpm, 4 °C, 10 min), 2 ml was mixed subsequently with 2 ml of each ninhydrin reagent and glacial acetic acid. This reaction mixture was heated at 100 °C for 1 h and cooled on ice, followed by the addition to it of 4 ml toluene. Brief vortexing and then stilling (15 min) of this mixture developed a light pink chromophore layer, which was retrieved for spectrophotometry (OD_520_) against a toluene blank. The resulting values were plotted over a standard curve using a commercially purchased proline (Himedia, Mumbai, India) to estimate proline concentration in the test samples.

#### Total chlorophyll and carotenoids

As per a standard protocol^122^, 100 mg of shoot biomass was crushed in 2 ml DMSO, and supernatant derived after centrifugation (at 5000 rpm, 4 °C, 15 min) was spectrophotometrically read respectively for chlorophyll a (OD_665_), chlorophyll b (OD_649_) and carotenoids (OD_480_). The resultant concentrations were computed and expressed into mg pigment per gram fresh weight as per recommendations^123^.

#### Total phenols and flavonoids

Respective samples of shoot biomass were subjected to air drying (24 h., 35 °C), and from each ca. 200 mg was crushed and mixed with 5 ml of 100% methanol. Supernatants were retrieved post centrifugation (10,000 rpm, 15 min, 4 °C) and stored for further use. Protocols each for phenol and carotenoid estimations observed slight modifications to methods referred elsewhere^124^. For estimating total phenols, 1 ml of this supernatant from each plant-derived sample and separate 1 ml aliquots each corresponding to a gallic acid standard (ranging from 10–100 µg ml^−1^) were individually resuspended into 5 ml SDW. Thereafter, 0.5 ml of Folin-Ciocalteu's reagent was added to each solution and mixed by shaking. By 5 min later, these were added with 1.5 ml of 20% sodium carbonate with finally volumetric makeup to 10 ml using SDW. Post incubation for 2 h at RT, blue color developed in each reaction vessel which was read spectrophotometrically (OD_750_). Resultant total phenols in tests were derived from the gallic acid standard plot and were expressed as mg of gallic acid equivalents per gram of dry biomass. For total flavonoids, as before 1 ml of supernatant from methanol extract of each plant sample and separate 1 ml aliquots, each corresponding to a quercetin standard (ranging from 100 to 1000 µg ml^−1^) were individually resuspended into 4 ml SDW. To these, 0.3 ml of 5% sodium nitrate was mixed and kept for 5 min at RT. Thereafter, 0.3 ml of 10% aluminium chloride was added to these. Abruptly at the 6th min, 2 ml of 1 M NaOH was added to each of the reactions, all of which then were volumetrically adjusted to 10 ml using SDW. A yellowish-orange color developed in each reaction vessel, which was read spectrophotometrically (OD_510_). The resultant total flavonoids in tests were derived from the quercetin standard plot and were expressed as mg of quercetin equivalents per gram of dry biomass.

### Statistical and computational analyses

All biochemical assays and experimental trials were performed thrice with at least 3 replicates per test (if otherwise, have been mentioned respective to experiments in relevant sections). The resulting data values for measurements were expressed as means of the three measurements with standard deviations (± standard deviation SD in tables, and/or depicted as error bars in graphs). For DNA barcoding analyses widely used software suits and/or programs and online applications were employed viz., CLC workbench (version 6.5.1), DECIPHER (version 2.19.2)^125^, VECSCREEN (https://www.ncbi.nlm.nih.gov/tools/vecscreen/), and NCBI BLAST (https://blast.ncbi.nlm.nih.gov/Blast.cgi). FTIR data was analyzed using Essential FTIR^®^ Spectroscopy Software Toolbox (https://www.essentialftir.com/index.html), graphs were prepared in MS excel and panel figures were assembled in MS PowerPoint.

## Results and discussions

### Isolation of JAS1 from ST PTC leaf explants

**Figure 1:**
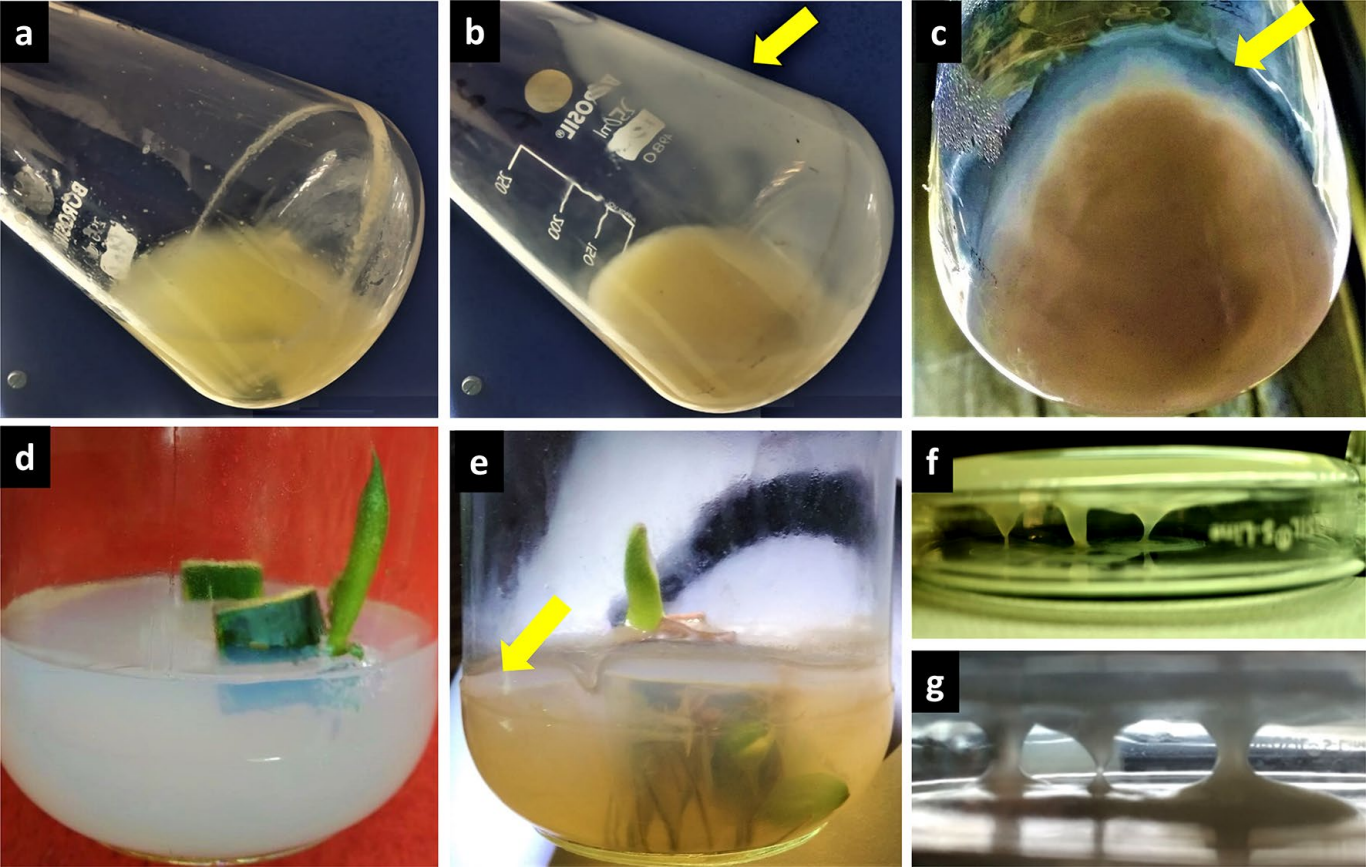
EPS secreting bacterial endophyte from ST leaf tissue cultures. ST leaf explants cultured over MSB normally generate callus suspension shown in (**a)**, but also unusually exhibited bacterial growth**,** which leaves a viscous material receding very slowly from the walls of the vessel upon tilting as shown in (**b,c)** (indicated with a yellow arrow); On MSA, in (**d),** a standard ST leaf explant led tissue culture**,** which often indicated profuse bacterial growth over the agar as in (**e)** (indicated with arrow); in (**f,g)** bacterial isolate over MSA produced enormous EPS drooping over the lid with time (plate inverted during incubations). All images represent results from various instances of ST PTC.

In our aseptic plant tissue culture facility, we routinely attempt callus induction and other whole plant regeneration trials with ST leaf segments. We recently reported a protocol for quick and efficient whole plant regeneration using IBA rooted leaf explants cultured at relatively high-temperature incubations^88^. In many of these and other trials, alongside the growth of intact callus, callus suspension, and the differentiating meristemoids, we also recorded 5–15% of our culture replicates showing extensive growth of some bacteria. On liquid media (MSB) it exhibited a peculiar highly viscous material sticking to the walls of the PTC vessels (Fig. 1a–c) while on semisolid media (MSA) it showed a distinct oozy bacterial growth (Fig. 1d,e). These did not negatively influence the in vitro plant propagation but favored morphogenesis with more root and shoot primordia over the ST explant (see further). Upon subculture, to MSA the isolate reproduced colonies overgrowing with time which drooped on the lid (Fig. 1f,g). From these instances as well as following a standard endophyte isolation protocol^89^ over healthy ST leaf segments, we could isolate similarly morphed bacterial clones and scrutinized them variously for their growth, molecular identity, and other characteristics following standard assays (discussed ahead).

### Biochemical and enzyme activity assays

Based on the morphological and biochemical characterizations (Table S1, supplementary file) the isolate JAS1 is a gram-negative, rod-shaped bacteria and it tested positive for catalase, methyl red, indole, and motility assays. Its ferments various carbohydrate substrates except lactose and gelatin and can limitedly grow in NaCl supplementation of up to 1%. Endophytes are known to produce and secrete various extracellular enzymes which significantly regulate key processes viz., nutrient acquisition, motility, symbiosis, commensalism, pathogenesis, etc. JAS1, however, tested positive only for protease production while remaining negative for pectinase, lipase, cellulase, and amylase. This may supportively hint at JAS1 co-cultivating with ST in vitro without influencing plant growth per se negatively testifying for enzymes known to degrade essential plant cell wall components such as cellulose and starch. It was intriguing otherwise as to why only protease activity could be detected in assays. Literature supports many of the endophytes positive for proteases implicated in equipping with extended antimicrobial defense^126^. We further plan to explore these effects in JAS1.

### In vitro growth of JAS1: EPS secretion, swimming, and swarming motility

**Figure 2:**
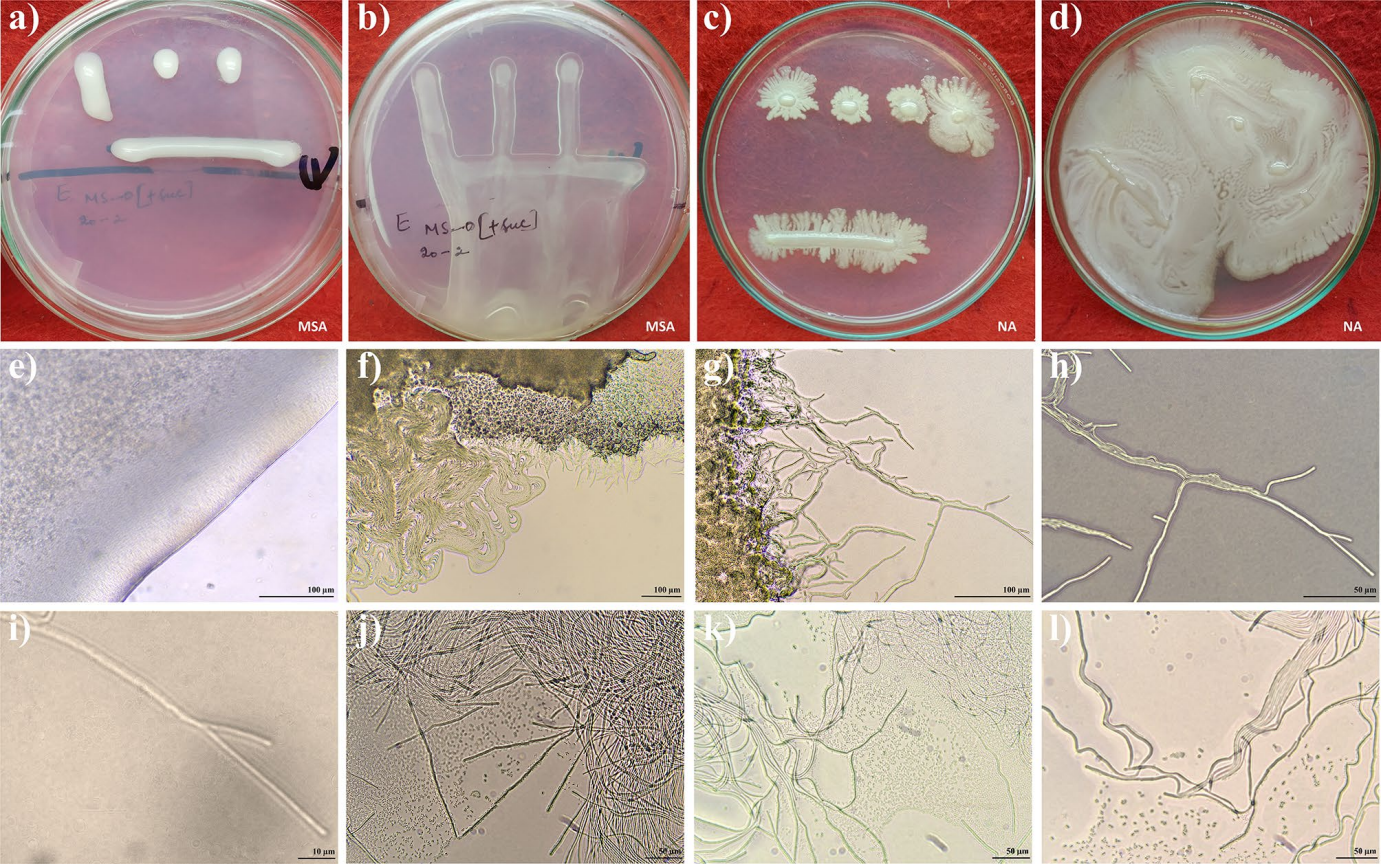
Growth characteristics of JAS1 on MSA and NA. In (**a–d),** dot and streak inoculated JAS1 over MSA (**a,b**) and NA (**c,d**). JAS1 in (**a)** depicts oozy/bulged growth (in 2 days’ culture) which in (**b)** droops underneath (by the fourth day in upright oriented plates). In (**c)**, JAS1 inoculation develops swarms (in 2 days) which in (**d)** covers the entire plate by the 3rd day. Panels (**e–l)** show light microscopy assisted close views of bacterial growth patterns on MSA (only **e**) or NA (**f–l**). In (**e)** shows an EPS front following right after the lane with higher bacterial density, however in (**f–l)** is shown the growth of JAS1 forming swarms, In (**f**), shown three superposed layers or fronts with different bacterial (density) patterns, wherein the upper-front with probably the older and excessively denser growth, followed then with the approaching middle-front with newly produced swarms (with spindles pointing outwards, probably yet to form a spiral and wavy swarms), and then the lower-front with well-formed snake-like wavy swarms (with spiral spindles in tufts); Of these the middle front is magnified sequentially (in **g–i**) to show the quorum spindles. In (**j–l**), well-formed wavy swarms with spindles (as the lower front in **f**) when mechanically disturbed show bacteria dislodged from the spindle formation.

Within two days of growth on MSA, the streak plated inoculum of JAS1 formed pale white irregularly shaped colonies with a peculiar bulge or ooze which on further incubation drooped over the petri-dish lid (Fig. 2a,b). Streaks on the NA plate, however, grew to form peculiar fingerlike outgrowths covering the entire media surface within two days post-inoculation (Fig. 2c,d). These projections characterize a multicellularity trait called ‘swarming motility’ witnessed, in some bacteria under certain in vitro conditions of growth^127^. The motility of JAS1 isolate was priorly confirmed using two standard methods. On semi-solid SIM Medium, it grew along the visible stab line rendering cloudiness in the butt. Alternatively, under light microscopy with wet mounts, swimming motility in discreet directions was evidenced. Oozy growth (as on MSA) is commonly seen with rhizobacteria and is known to effectuate from the synthesis and secretion of certain exopolysaccharide (EPS) complexes. EPS synthesis is influenced majorly by both the concentration and types of sugars and nitrogenous species in the media^128^ as also witnessed for JAS1 (data not shown). EPS complexes may render such bacteria to develop mucoid (or slimy) and ropy colonies^129^. JAS1 however indicated only slimy EPS secretion on MSA (Table S1, supplementary file). Hence, the JAS1 isolate showed apparent variability in growth patterns on two different media, which respectively reveal its EPS production and swarming motility features.

Further, under the light microscope the JAS1 growth on both MSA and NA was closely observed (Fig. 2e–l). Compared to that on MSA, where the streak margins always exhibited a peculiar EPS front backed by a population gradient of JAS1 (Fig. 2e), the swarm margins on NA however showed distinguished formations (Fig. 2f). Further on NA, most of the emerging swarm outgrowths appeared exclusively in wavy and/or spiral fashion. Each of the waves or spiral assemblies showed a well-defined length within the swarms (Fig. 2g–i). Freely moving bacteria were more apparent at the swarm fronts than in the back (Supplementary Video 1). Upon disturbing some of the bundled spirals by gently pressing with a fine aseptic needle, which probably dislodged individual bacterium off the spirals (Fig. 2j–l). Dislodged bacteria surprisingly showcased their swimming motility over the area (notched wet) with the pressed agar (Supplementary Video 2). These observations indicate that JAS1 equips both swarming and swimming motility which might be variously showcased in vitro depending on the media composition and substratum factors. Such growth and motility attributes have been shown in many bacteria including as well AP strains^38,130^. Swarming also exemplifies quorum sensing in bacteria and helps bacteria to variously cling/attach facilitating their easy and speedy escape from local stresses, migration to favored and preferred localities, and/or the efficient invasion of the host^127^. In some plant-associated bacteria, swarming motility is known to aid virulence while other many are implicated in use as biocontrol agents as they deliver protection to their plant host(s) from other competing bacterial and fungal phytopathogens^127^. Both swarming and swimming activities facilitate root attachment, and colonization^131,132^. Answers to how JAS1 orchestrates these features, especially in light of its PGP attributes (discussed ahead) demand further studies.

### Molecular identification of JAS1

**Figure 3:**
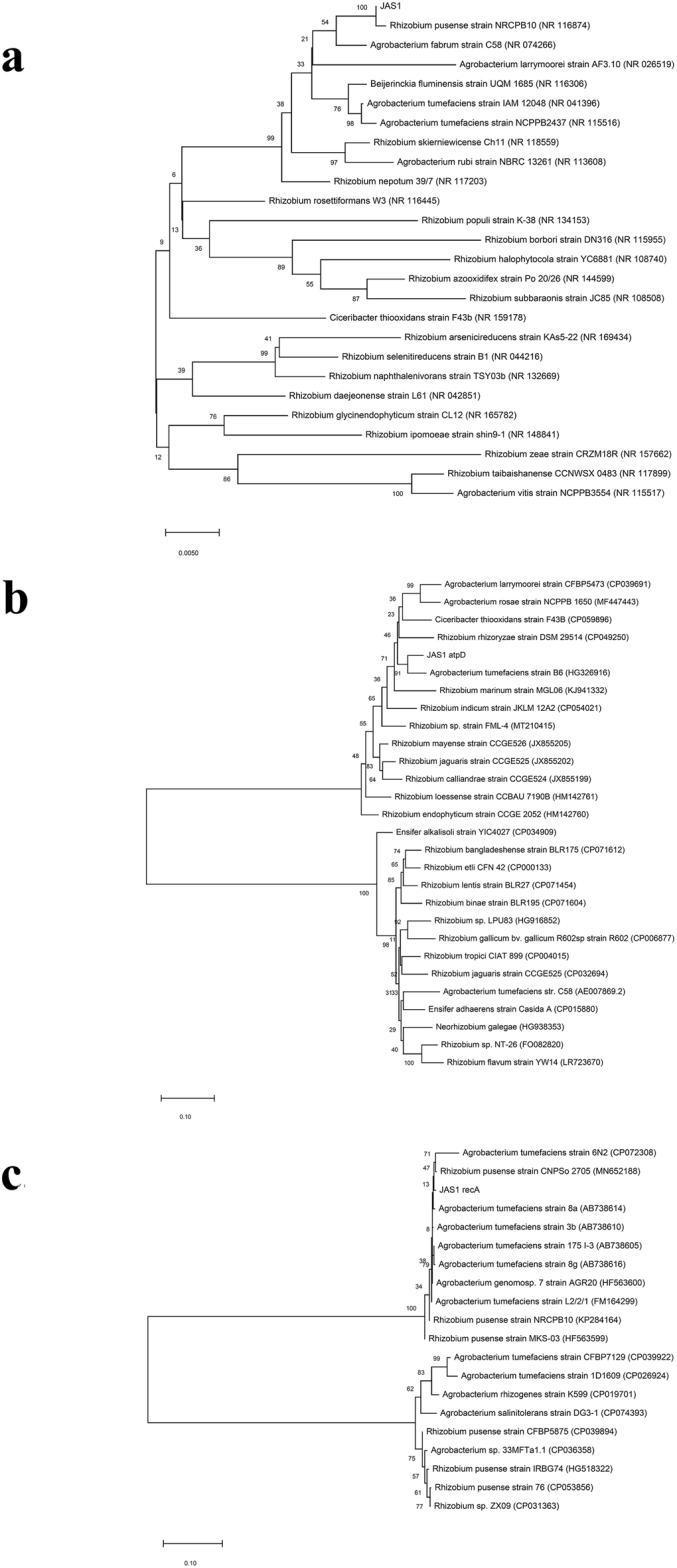
Phylogenetic relationship of JAS1 with closely related bacteria. Evolutionarily related strains shown with neighbor-joining tree based on gene sequences as in (**a)** for 16S rRNA (1396 bases; partial cds); in (**b)** for *atpD* (1455 bases; full cds); and in (**c**), for *recA* (1089 bases; full cds). Numbers specify bootstrap values based on 1000 resamplings. Bars in each tree scaled to nucleotide substitutions per position, respectively. Accession numbers are mentioned within braces.

We performed molecular identification of the bacterial isolate by comparing 16S rRNA, *recA,* and *atpD* gene sequences. Partially resolved 16S rRNA sequence (1396 bases) in JAS1 (Genbank accession number MW827601), showed high similarity (99.64%) with the *Rhizobium* *pusense* strain NRCPB10^T^ (GenBank accession number NR_116874), followed by *Beijerinckia* *fluminensis* strain UQM 1685 (99.43%), *Agrobacterium* *fabrum* strain C58 (99.36%), *A.* *tumefaciens* strains IAM 12048 (99.13%), and NCPPB2437 (99.00%). The phylogenetic tree revealed close relatedness of JAS1 with recognized *Rhizobium* *pusense* strain NRCPB10^T^ (GenBank accession number NR_116874)^30^ as both share a common ancestor (Fig. 3a). These results confidently position JAS1 within the genus *Agrobacterium* (considering as well the revised inclusion of *Rhizobium* *pusense* strains into agrobacteria)^31,32^*.* Further, we deduced the complete sequences of two housekeeping genes (*atpD* and *recA*) PCR amplified from JAS1 (GenBank accessions MZ741443 and MZ741444 respectively), and analyzed their phylogenetic relatedness with other known species, after multiple sequence alignment. Based on sequence alignments, *atpD* in JAS1 could only sparsely relate to a few *A.* *tumefaciens* strains (B6, 93.89%; and C58, 93.33%) and *R.* *endophyticum* strain (CCGE2052, 91.03%), but surprisingly did not align to any of the reported *A.* *pusense* strains. Also, *Agrobacterium* strains showing maximum sequence similarity were distantly oriented in the phylogenetic tree (Fig. 3b). In *recA* (Fig. 3c), JAS1 relates more significantly to many *A.* *pusense* strains (CFBP5875, 99.63%; NRCPB10^T^, 99.54%; LMG25623^T^, 99.45%; IRBG74, 99.36%; CNPSo 2705, 99.35%; FDAARGOS_633, 99.17%; SX41, 99.17%; MKS-03, 99.16% and strain 76, 99.08% etc.) as well as to *A.* *tumefaciens* strains (L2/2/1, 99.79%; 8a, 99.38%; 3b, 99.28%; 175 I-3, 99.17%; and 8 g, 99.07%). Overall, the complete coding sequence of JAS1-RecA did not align completely with any agrobacterial strains. Notably, even with 79% of its sequence coverage, it still could not align identically with the typed strain NRCPB10^T^ (99.54% sequence identity). These results thus largely infer that JAS1 is a novel strain *A.* *pusense* which probably had co-evolved with/from other agrobacterial species.

### Antibiotic sensitivity tests

JAS1 was found sensitive to many tested antibiotics (Table S2, supplementary file). In respect to those reported for the *A.* *pusense* typed strain, NRCPB10^T^^30^, it differed in showing resistance only to ampicillin, and trimethoprim while being susceptible to nalidixic acid, tetracycline, rifampicin, neomycin, kanamycin, streptomycin, and spectinomycin.

### EPS production, purification, and total yield

EPS from various microbial sources have found multifaceted applications^133,134^ and have been variously studied considering EPS biosynthetic potential of each strain(s) and the influence over it from various parameters viz., physical factors, media components especially the carbohydrate and nitrogenous sources, use of elicitors and so on^129,135,136^. EPS yield also depends on the extent of its recovery from losses in various downstream extraction and purification processes, all of which vary from simple centrifugation to complex hydrolysis, precipitation, and other recovery steps. At the time of writing the manuscript, no elaborate studies could be found documenting EPS production amongst the known AP strains, except that of a commercial β-glucan (marketed as Salecan) released by the strain ZX09^137^. Other AP strains were limitedly reported to date with mucoid colonies and/or milky white streaks on agar media.

Our chanceful findings with the strain JAS1 hail from its enormous EPS secretion in PTC media which mandatorily contains 3% sucrose. We manifested a simple approach of diluting the resulting culture volume enough to separate cell biomass using centrifugation followed by ultrafiltration to assure cell-free, near pure extraction. Additionally, to cater to purity requisites in FTIR and SEM (next sections) we further dialyzed and lyophilized EPS preparations. On average, 10 days’ old culture of JAS1 spread plated on 20 ml semi-solid media MSA over a 100 mm diameter petri-dish yielded ca. 56 ± 2 mgs of EPS. Small scaled batch culture in 50 ml of MSB inoculated with JAS1 for 10 days offered a greater EPS yield of ca. 360 ± 2 mg. Surprisingly, however, batch cultures of JAS1 in a liter volume of MSB within the same duration resulted in an averaged total recoverable EPS yield of 7252 ± 2 mg l^−1^. This is a fairly appreciable yield reported over that known from the AP strain ZX09^137^ and is also comparable to that from other EPS-producing *Agrobacterium* species. Yield from the small scale (50 ml) batch correlated well with that resulting from a liter’s scale interpreting an as minimal loss of only about 14 mg during the downstream processing with scaled-up produce.

### FTIR and XRD analysis of EPS

**Figure 4:**
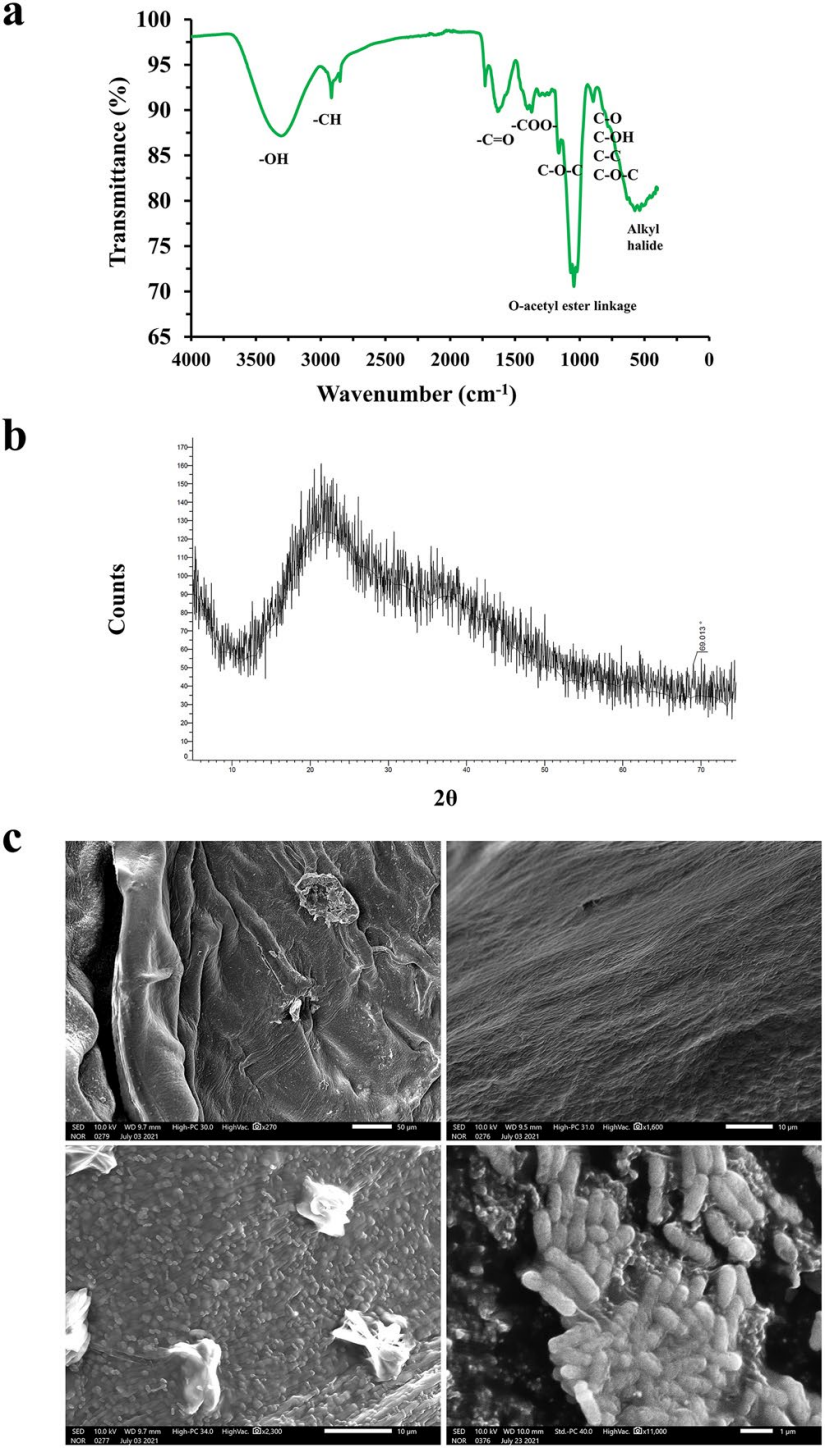
FTIR, XRD, and SEM analyses. In (**a**) FTIR of lyophilized EPS released from JAS1 studied in the range of 4000–400 cm^−1^, In (**b)** its XRD of EPS, while in (**c)** SEM micrographs of EPS (in above two and lower left panel) and JAS1 isolate shown bathed in EPS (lower right panel).

The functional groups of EPS-JAS1 were studied with the use of ATR-FTIR (Fig. 4a) which depicted an intense broad peak at 3306.21 cm^−1^ corresponding to the presence of hydroxyl groups^138^. A small band witnessed at 2916.88 cm^−1^ unique to that for carbohydrates infers the presence of asymmetric C–H stretching vibration^139^. Furthermore, an asymmetric stretching peak was observed at 1632.92 cm^−1^, corresponding to stretching vibrations from carbonyl groups (C=O) in CONH moieties^140^ and may also interpret ring stretching vibrations in galactose and mannose^141^. The bands detected at 1416.03 cm^−1^ detects symmetrical stretching of COO− groups^138^. The smaller peaks at the 1267.07 cm^−1^ band are due to C–O stretching vibrations relevant to free carboxylic acids^142^. Absorption peaks around 800 and 1200 cm^−1^ would derive from sugars as well as β-glycosidic bonds among their monomers. The absorption peak at 1046.19 cm^−1^ is designated to C–O–H, C–O–C, and C–O, inferring polysaccharides in the sample^140,141,143^. The peaks at 895.44 and 815.97 cm^−1^ correspondingly indicate the α-glycosidic and β-glycosidic bonds^144^. The small peaks observed at 559.11 and 623.55 cm^−1^ depict glycosidic linkages in the polysaccharide^145^. The absorption peaks between 550 and 539 cm^−1^ match with stretching vibrations of the alkyl-halide groups^143^.

The appearance of these functional groups enables JAS1-produced EPS (JAS1-EPS) to find potential use in numerous applications. For example, the water-loving nature of EPS is attributed to the presence of hydroxyls^146^ which vouches the emulsifying^138^ or biosurfactant property^147^ for this EPS. Carboxylate groups ensure affinity to opposite charge molecules such as heavy metals^148^. The anionic nature and ability to chelate metals and ions ensure anticorrosive effects when applied to metals^149^. The above findings on functional groups from EPS secreted by JAS1 were found almost similar to those mentioned for exopolysaccharides called curdlan known to be secreted by many bacteria^150–152^. Phase identification of materials is purposefully gathered using XRD. XRD pattern of EPS (Fig. 4b) with broad peaks revealed prominent amorphous nature (83.4%) and sharp narrow peaks depicted low crystallinity (16.6%) profile.

### SEM of EPS and JAS1

SEM has been used to account for surface characteristics of polymers^153^. SEM micrographs depict that EPS from JAS1 has both smooth and rough patches at discreet locations on its surface exhibiting as well with irregular folds (Fig. 4c) which may impart compactness to the otherwise dispersive configuration. Small and variously sized pores were also witnessed which (along with folds) may suffice permeability and water-holding features qualifying JAS1-EPS for use in viscosifiers, thickeners, and preservatives for new-age foods^154^. Porosity may also help bacterial dynamics in plants, soil, and other factors as well within biofilms^155,156^. JAS1 can be seen as peculiar rods bathing in EPS (Fig. 4c).

### EPS solubility in different solvents

Dried exopolysaccharide was found completely soluble in water availing a homogenous and transparent solution with concentration-dependent increments in viscosity. EPS was however found insoluble in other tested solvents viz., ethanol, methanol, butanol, ethanediol; acetone, ethyl acetate, DMSO, chloroform, hexane, and benzene. These results are in congruence with the FTIR results and those reported for EPS known elsewhere, supportive in that its solubility exclusively in aqueous solvent correlates with the predominant hydroxyl groups in EPS^157–159^.

### Plant pathogenicity testing of JAS1

As mentioned before, JAS1 occurred in many of our PTC attempts with ST leaf explants where it did not negatively affect the vegetative growth of ST. However, many species in the agrobacterial genera are reported as plant pathogens while also some exist as symbionts and/or commensals in their host(s). Surprisingly, plant pathogenicity amongst AP strains has only recently been reported from Lawson Cyprus tree isolate, KH1^104^, known to cause tumors over carrot root discs^104^. Upon similar tests, JAS1 however failed to indicate any pathological symptoms on carrot root discs. Unfortunately, such pathogenicity assessments have not been made for other known AP strains as also only a few of these are studied for their PGP attributes. Some clinically reported AP strains are also known to cause sepsis in humans^160^, which warrants safety assessments over proclaimed PGP AP strains, their finished bioproducts (as also on those many known from various other microbes) including as well EPS, many have seen commercial translations with the consideration of generally regarded as safe (GRAS)^161^. Studies elsewhere as well as ours hint that AP strains may have evolved multiply and independently in natural extremities to showcase either a PGP and/or otherwise a pathogenic behavior.

### PGP characterizations

#### Phosphate solubilization

Phosphate is an important but also most limiting macronutrient in soil, with poor bioavailability to plants due to its insoluble forms mostly occurring as rock phosphates. To quench the deficit, most plants rely on endophytes capable of phosphate solubilization by soil acidification^162^. JAS1 was detected positive for phosphate solubilization, with a peculiar halo zone developed around its colonies on Pikovskaya’s agar and a phosphate solubilization index (PSI) of 1.27 cm on the 9th day post-inoculation (Table 1). The highest solubilization potential (17.6 µg ml^−1^) could be achieved within 48 h in liquid NBRIP medium. Like JAS1, another AP strain MB-17a isolated from mung bean roots is also reported with slightly higher phosphate solubilization (PSI of 2.67 cm and solubilization potential of up to 53 μg ml^−1^ on the 6th day)^33^, while two other studied isolates from soybean and tomato are reported negative for phosphate solubilization potential^38,43^.

**Table 1:** Plant growth promotion assays.

PGP attributes	48 h	120 h	160 h	216 h
Phosphate solubilization index (cm)	–	–	–	**1.27 ± 0.03**
Soluble P when TCP is a substrate (µg ml^−1^)	**17.6 ± 0.14**	13.05 ± 0.03	12.37 ± 0.05	11.59 ± 0.13
Siderophore production (%)	–	**30.1 ± 0.56**	–	–
Ammonia production (µmol ml^−1^)	39.7 ± 0.14	**42.66 ± 0.15**	33.05 ± 0.02	31.65 ± 0.13
Production of auxin (IAA) (µg ml^−1^)	68.02 ± 0.49	77.13 ± 0.35	**86.95 ± 0.49**	64.46 ± 0.21
Gibberellic acid production (µg ml^−1^)	95.79 ± 0.85	**172.98 ± 1.86**	136.26 ± 0.54	130.71 ± 0.54
Zinc solubilization index (cm)	–	–	1.35 ± 0.07	**2.3 ± 0.15**
Zinc solubilization (µg ml^−1^)	120.33 ± 0.33	131.66 ± 0.88	**193.67 ± 0.88**	153 ± 0.57
Potassium solubilization	–	–	–	–
*ACC* *deaminase* production	+	+	+	+
Nitrogen fixation	+	+	+	+
Hydrogen cyanide production	–	–	–	–

Observations as per standard assays were periodically recorded and the highest values of effects are highlighted in bold. Values represent the means of three replicates in each of the three independent trials.

#### *ACC deaminase* production

1-aminocyclopropane-1-carboxylic acid (ACC) is a precursor of ethylene, the phytohormone that under low titers enhances seed germination, root length, and root hair growth^163^. The successful growth of JAS1 tested over DF media inferred positive *ACC* *deaminase* production of (Table 1). This PGP screen evaluates the ability of bacteria to control deleterious ethylene levels allowing their plant hosts to better manage various biotic and abiotic stressors^163,164^. *ACC* *deaminase* is reported in three other AP strains viz., MB-17a, RJG6, and IC59^33,34,165^.

#### Siderophore production

Siderophores are secondary metabolites with low molecular weight and have iron-chelating potential^166^. It is involved in growth and development, and in restricting the pathogens proliferating in plants. JAS1 showed a peculiar yellow halo zone around its streaks over CAS agar inferring a positive siderophore production profile, quantifiably of 30.1% (%SU) at the 120^th^ hour of growth (Table 1). Previously, the IC59 strain of AP was reported with siderophore production of up to 30.6% units^34^ while few others viz., YP3, YP4, MB-17a, and MS-1 strains were reported positive for siderophores^40,43^.

#### Indole-3-acetic acid (IAA) production

IAA is an important auxin that principally enhances root growth in plants^167^. JAS1 displayed appreciable IAA production (max. 86.95 µg ml^−1^) as determined by the Salkowski assay (Table 1) and is comparable to that in the AP strain MB-17a (reportedly producing 110.5 µg/ml IAA)^33^. Other reported AP strains such as YP3, YP4 and IC59 produced low IAA (59.2, 43.8, and 21.9 µg ml^−1^, respectively)^34,40^, and few others were reported merely as positive for this PGP trait^38,40,43^.

#### Ammonia production

Ammonia variously sequesters plant growth development by a build-up of soil assimilable nitrogen resources, inhibition of pathogen attack, and biomass production among others^168,169^. JAS1 was found positively producing ammonia with the maximum release (42.66 ± 0.15 µmol ml^−1^) detected on the 5th day of assay incubations (Table 1). Only a few other AP strains viz., MB-17a, AM-4, and MS-1 have been tested positive for this attribute.

#### Gibberellic acid production

GA regulates root and stem growth and also improves seed germination in plants^170^. The highest GA production of 172.98 µg ml^−1^ was seen by JAS1 at the 120th h of the incubation regime (Table 1). This property has not been ever reported for any AP strain.

#### Zinc solubilization

Zinc deficiency in plants is considered a critical factor limiting crop productivity. Its sensitivity is more pronounced during drought stress and studies as well suggest that water use efficiency is critically dependent on plants’ zinc profile^171^. Many soil zinc forms occur in complex with oxides, silicates, and carbonates while microorganisms are reported to augment soil fertility by solubilizing these forms^172^. Zinc solubilization effect JAS1 was preliminarily ascertained over insoluble zinc oxide in plate and broth assay. In plate assay, a zinc solubilization index of 2.3 cm was recorded at the 216th h of incubation, which continued to increase beyond the observation schedule (Table 1). This apparent finding was also confirmed quantitatively by AAS wherein JAS1 could offer almost 200 μg ml^−1^ week^−1^ solubilization of insoluble ZnO. This PGP trait is reported for only one AP strain IHCP2 solely using the plate method inferring a lower solubilization index (1.87 cm)^39^.

#### Potassium solubilization

JAS1 failed to detect any potassium solubilization, the latter for which few other AP isolates have been reported positive viz., stains 59, L3/4, OPVS03, and OPVS06^37,173,174^.

#### Nitrogen fixation

Nitrogen is a vital nutrient for organismic growth. However, plants are unable to utilize atmospheric ample of nascent nitrogen and for the supply of its assimilable forms rely on certain nitrogen-fixing microbes^175^. N_2_-fixing bacteria thus can grow over nitrogen-free media in vitro. Our JAS1 isolate showed growth over nitrogen-free Jensen’s media and showcased glistening colonies 7 days post-inoculation (Table 1), thus indicating a positive N_2_-fixing PGP trait. Similarly, TMV2-6, YP3, YP4, and PR1 strains of AP are also known to confer this ability^40,176^.

#### Hydrogen cyanide production

Hydrogen cyanide is a volatile secondary metabolite synthesized by several PGPRs and is implicated as a biocontrol agent causing cytotoxic death of various plant pathogens^177^. However, the JAS1 isolate did not show HCN production ability. This trait is reported in only a few AP strains^34,37,39,43^. Recently, a group had shown that HCN production does not necessarily suppress plant pathogens, however, suffices phosphate availability in soil^178^. Given this, the JAS1 isolate already shows phosphate solubility trait (discussed before) while lacking the HCN production ability. This might suggest that HCN production and phosphate solubilization traits are independently governed in PGPRs, however, this needs extensive studies.

#### Biofilm formation

The biofilm formation (BF) property of JAS1 was assayed using the Congo red agar method. The isolated strain JAS1 showed strong BF after 24 h (Table 1) and produced black-colored colonies with a dry consistency. BF could be obviated already during sampling of JAS1 isolate in MSB cultures of ST depicting the biomass aggregates attached to the interior of the flasks forming a thin lining of JAS1 (Fig. 1). This BF is due to the EPS, as generally reported in many plant-associated bacteria which helps in adhesion to plant surfaces, and mitigating various abiotic and biotic stresses^23,179–181^.

### Effects of JAS1 on ST regeneration in vitro

**Figure 5:**
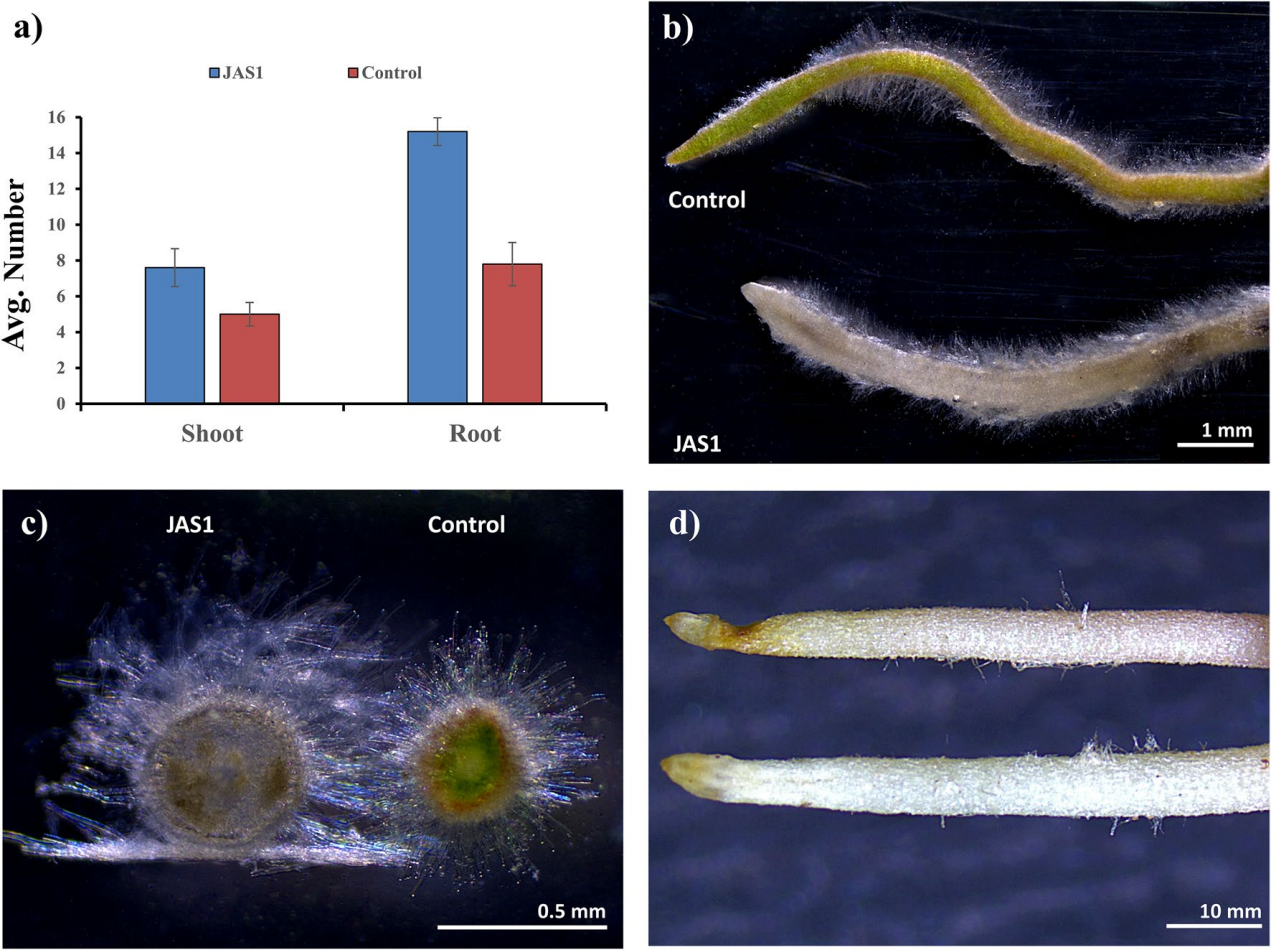
Effects of JAS1 on ST regeneration in vitro. In (**a)**, increments in number of shoot and root primordia; in (**b)**, change in root texture; in (**c)** root girth and root hair length; and in (**d)** field grown ST roots. Panels (**b–d**) show stereomicroscopic views with scaled bars.

We tested the effect of bacterial isolate JAS1 on in vitro rooted ST leaf explants using our previously standardized PTC protocol for ST whole plant regeneration^88^. After 5 weeks, the JAS1 co-cultivated explants resulted in significantly enhanced shoot and root growth responses (Fig. 5a). In addition, roots in the JAS1 treatments showcased changes in texture from green to pale white (Fig. 5b), significant increments in girth, and lengthier root hairs compared to those in the control treatment (Fig. 5b,c). Roots from field-grown ST, however, did not exhibit these observations (Fig. 5d). These comparisons support that PGP prospects with JAS1 were delivered to its host plant (ST) while it’s standardized in vitro regeneration regime. No pathological symptoms could be recorded in any of the replicates, all of which subsequently resulted in productive whole ST regenerants.

### JAS1 enhances ex vitro growth of wheat seedlings

**Figure 6:**
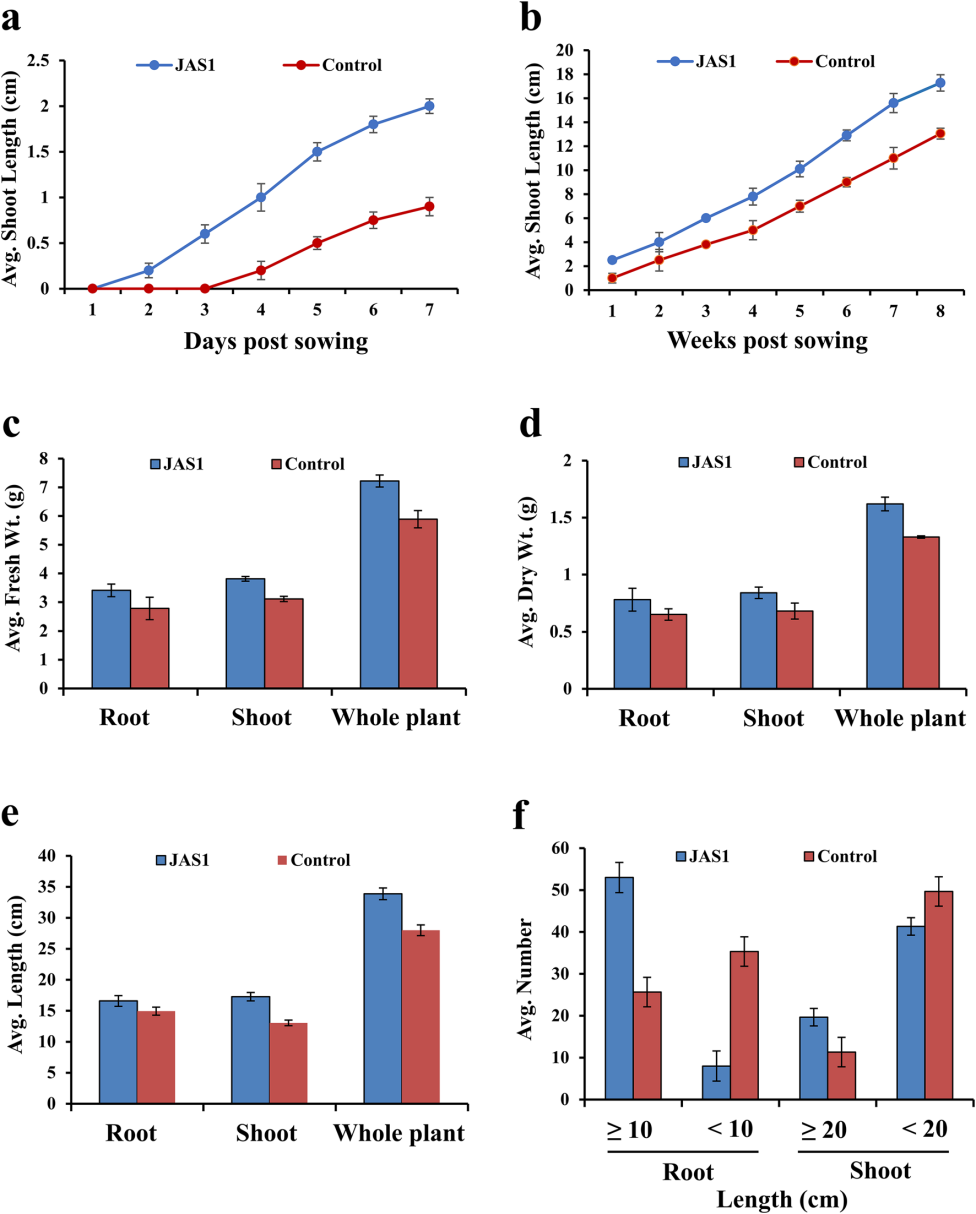
Effect of JAS1 on ex vitro grown wheat seedlings. In (**a,b)**, seedling emergence and shoot growth estimates respectively as length increments recorded with time. Data on wheat growth profiles shown in (**c–f)** derived post drain-outs at the end of 8 weeks’ trial(s). Length measured from root shoot interface adjoining the pod. Datasets represent the means of the means from three trials.

**Figure 7:**
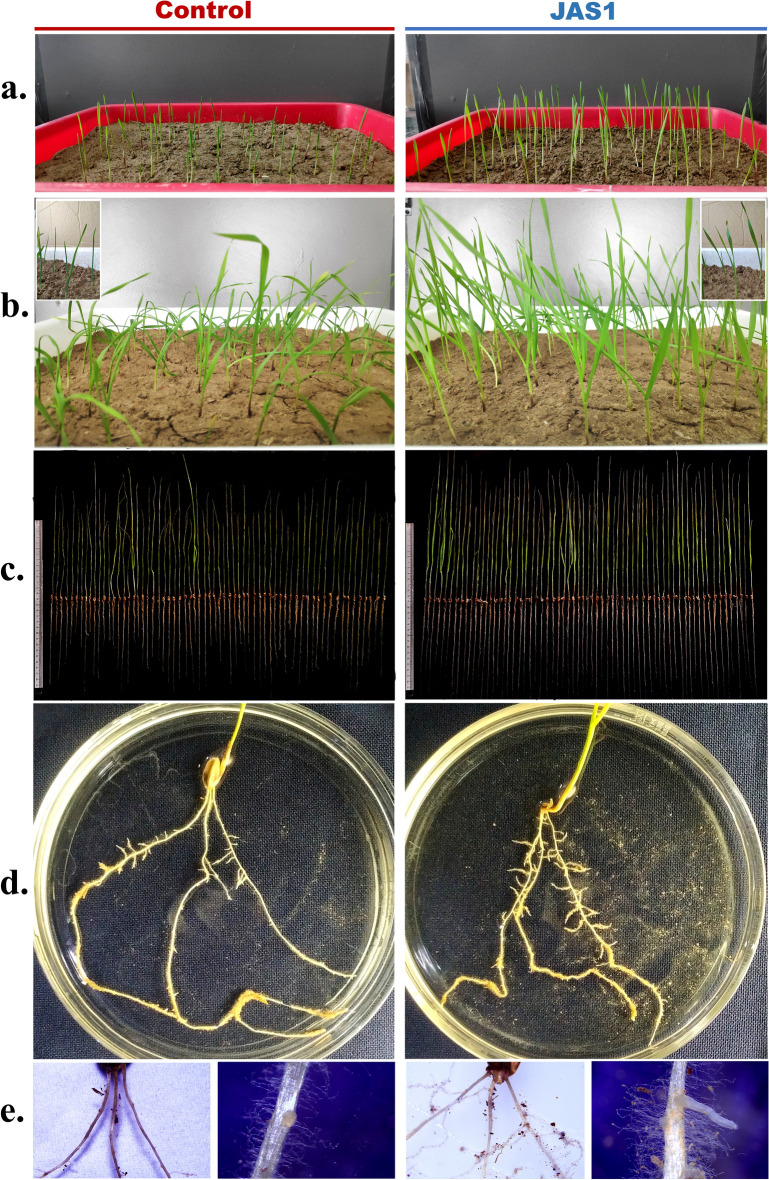
PGP effects of JAS1 observed on ex vitro grown wheat. All figures on the left represent control treatment while those on right correspond to JAS1 treatment trials. Shoots in (**a)** at 3 weeks; in (**b)** at 8 weeks (in insets at 5 weeks) post sowing. In (**c),** seedling profile post drain out after 8 weeks growth over well-watered soils. In (**d),** prominent root branching under JAS1 treatment, which in (**e)** shown well distinguished at close to the root-shoot interface. Also, in (**e)** prominent increments in root-hair density and length under JAS1 treatment. Roots in seedlings under JAS1 treatments exhibit soil aggregates eroding from the roots on swirling the plate (as in **d)**, otherwise remain attached (as in **e**).

We studied the growth performance of wheat seedlings under the effect of JAS1. This used a seed priming approach for a commercial wheat variety which germinated and grew over well-watered tray soil-beds, and was observed all through for 8 weeks. With respect to (*wrt*) the unprimed seeds in the control sets where shoot emergence was withheld up to the 4th day, the JAS1 treated seeds showed early germination with clear emergence of shoot primordia above the soil within 3 days of sowing (Fig. 6a). Seed germination enhancement is a common prospect of PGPRs probably as also seen here with JAS1. The shoot primordia sustained higher growth increments in lengths *wrt* those in control throughout the 8-week trials (Figs. 6a,b, 7a–c). Furthering germination, in these trials we observed other various effects from JAS1 that enhanced the wheat seedling developmental profiles. For example, at 5 weeks post sowing, the first leaf easily uncurled and split up from the seedlings (Fig. 7b insets), while this was delayed by ca. 1 week in the untreated control sets. Followed after this shoot in the JAS1 treated beds showed healthy upright stature than those in the control set(s), the latter which with a distinct bent and with older leaves showcasing a peculiar yellowing at their tips extending towards the base, however, without any signs of interveinal chlorosis (Fig. 7b). No such detriments could be recorded in any of the JAS1 treated test sets. Such leaf tip yellowing reportedly corresponds to a temporary nitrogen deficiency in wheat which either entails from the soil low in nitrogen and/or often its waterlogging^182^. Obviously, throughout these experimental trials, soil profiles were maintained equally well-watered and in addition, no seepage was provisioned. It is also to note that the soil beds with JAS1 primed seeds were booster dosed intermittently thrice. In addition, soil testing also indicated wheat-productive improvement of its fertility by JAS treatment (data not shown). This supports the successful translation of the PGP attributes of JAS1 in improving both soil and plant health. Data recorded post drain-out inferred that JAS1 treatment enhanced shoot and root biomass (Fig. 6c,d), and their lengths (Fig. 6e,f). In addition to this, comparative speculation of the root formations revealed that almost all roots in the JAS1 treatments had more branching points than on those in the untreated set (Fig. 7d), as well as that root branching is evident even at sites closer to the pod or that lay closer to topsoil profile (Fig. 7d,e). When observed under a stereomicroscopic magnification, roots from seedlings in the JAS1 treatment showcased denser and lengthier root hairs than those in the control treatments (Fig. 7e). Soil particles also adhered better to the roots under JAS1 treatment (Fig. 7d,e). Presumably, they form aggregates on the rhizosheath due to the viscous EPS secretion by JAS1 as also shown with many other rhizobacteria^183^. Enhanced branching, hair length, and density in roots are also established outcomes of PGPR application in plants.

### JAS1 maintained plant and soil health under intermittent soil drought

**Figure 8:**
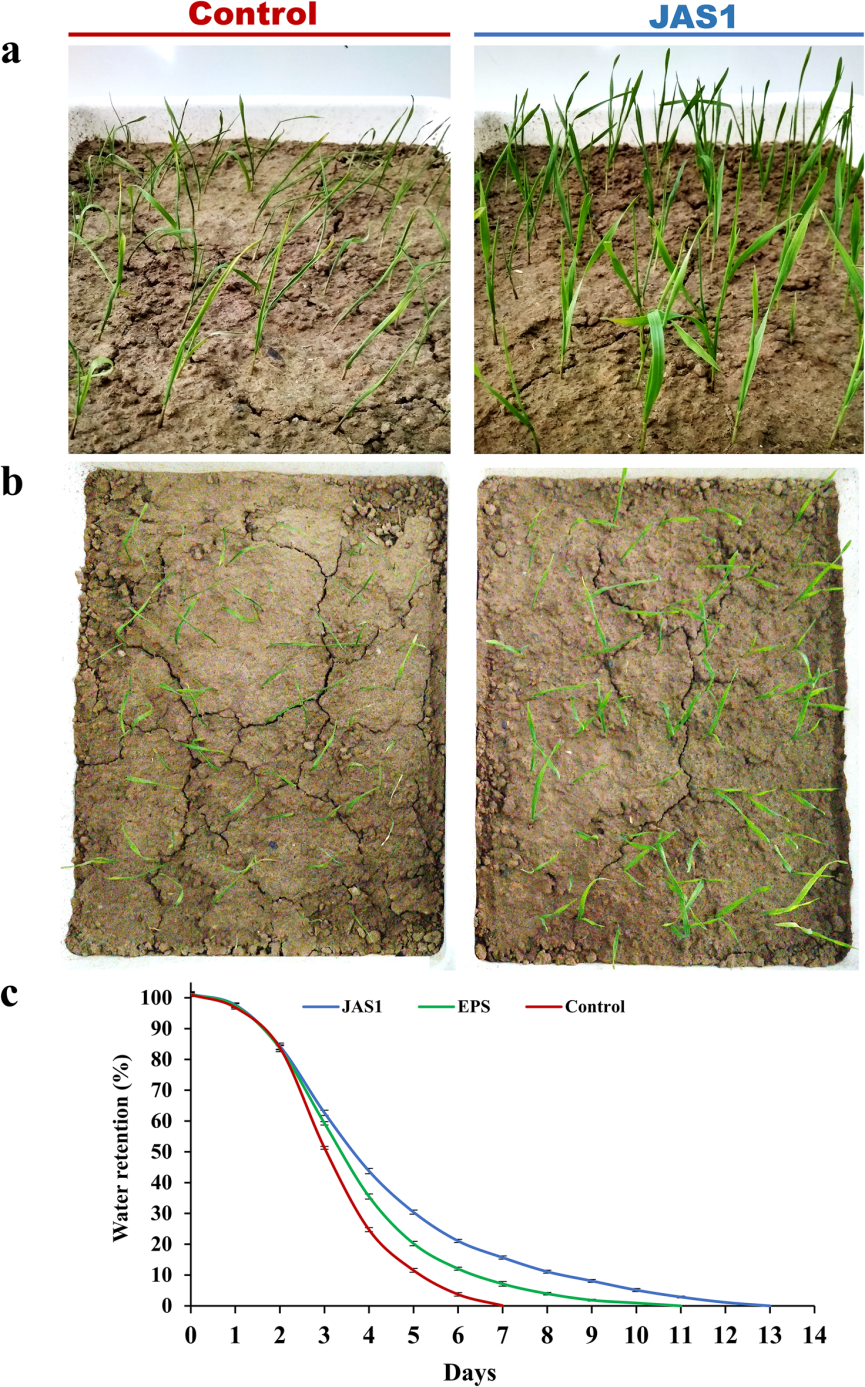
Effect of JAS1 on plant and soil health under a dry regime. In (**a)** wheat seedling performance, and in (**b)** the extent of soil cracking developed after 10 days’ soil drying. In (**c)** effect of JAS1 and its EPS over soil water retentions at periods under steady soil drying regime.

While maintaining the above experimental design we also separately tested the effect of intermittent soil drying over JAS1-treated seedling growth. Watering of soil beds was completely withheld post 6 weeks of sowing for 10 days. We witnessed gradual leaf squinting and drooping in shoots in the control sets (Fig. 8a) as opposed to their normal growth in the same period under well-watered regimes. Shoots in the JAS1 treatment trays however maintained their overall growth stature at all times till the 10 days (Fig. 8a). Upon rewatering most seedlings in the control sets could not regain the healthy stature but wilted while those in JAS1 treatment continued to propagate normally without any signs of growth detriments (data not shown). In addition to this, we also noted a peculiar soil cracking phenomenon, usually witnessed in farm fields under conditions of drought. Close speculation of the tray trials depicted that soil beds with JAS1 treatment had developed comparatively lesser cracking extent than the beds in the control treatment (Fig. 8b). These results indicate that JAS1 treatment of wheat could rescue plants from intermittent soil drought. EPS releasing PGPRs are known to protect plants from drought tolerance by the formation of rhizosheath around the roots and as well by enhancing the soil water uptake^183,184^.

These notions warranted testing the effect of JAS1 and its EPS on soil water retention. Soil beds prepared in plastic Petri-dishes were completely drenched with either MSB resuspended JAS1 pellet or water resuspended EPS or just water and were followed with a continuous drying regime. Under these trials, water-drenched soil beds showed complete drying within a week (Fig. 8c), however at this time point soils in the EPS and JAS1 treatment dishes still retained ca. 8% and 16% water and with their complete drying scenes delayed to ca. 11th and 13th day, respectively. These results indicate that JAS1 is effectively able to enhance water retention of soil and that water-soluble EPS released by JAS1 could be the possible governing factor. Soil cracking extent was well reduced in JAS1 and EPS treatment than in control treatment (data not shown).

These results support our notion of reduced soil cracking effect in JAS1-dosed soil beds. Higher water retention in JAS1 treated soil bed *wrt* that with EPS treatment was obviously due to the presence of MSB supportive to JAS1 growth and EPS secretion. The presence of MSB may be analogous to the presence of wheat plants in tray trials which might accordingly govern growth and EPS release in soil beds from JAS1 (dosed actually as water resuspended inoculum). Besides, positive growth and EPS secretion in JAS1 on agar solely supplemented with extracted ST, wheat biomass, and/or coconut water were also verified from other ongoing studies (data not shown). JAS1 growth and/or productive EPS release in soil beds otherwise treated exclusively with water-resuspended inoculum was negligible (data not shown).

The water solubility of the EPS may allow its expansive reach, deposition, and coverage as well as that of the releasing bacteria also abreast by various other mechanisms (bacterial swimming and swarming motility) to other portions within the soil profile. The ability of such bacteria to proliferate, within roots and/or on rhizoplane would allow extended cushioning besides the other PGP effects delivered consistently to the plant host, especially under abiotic stresses such as drought. In bigger field settings, how this may translate into crop productivity, and soil performance, otherwise also under various stressors is yet under testing for JAS1 (data not shown). Many PGP bacteria are already in commercial use as bioinoculants and biofertilizers^185–188^.

### JAS1 also enhances in vitro growth of wheat seedlings

**Figure 9:**
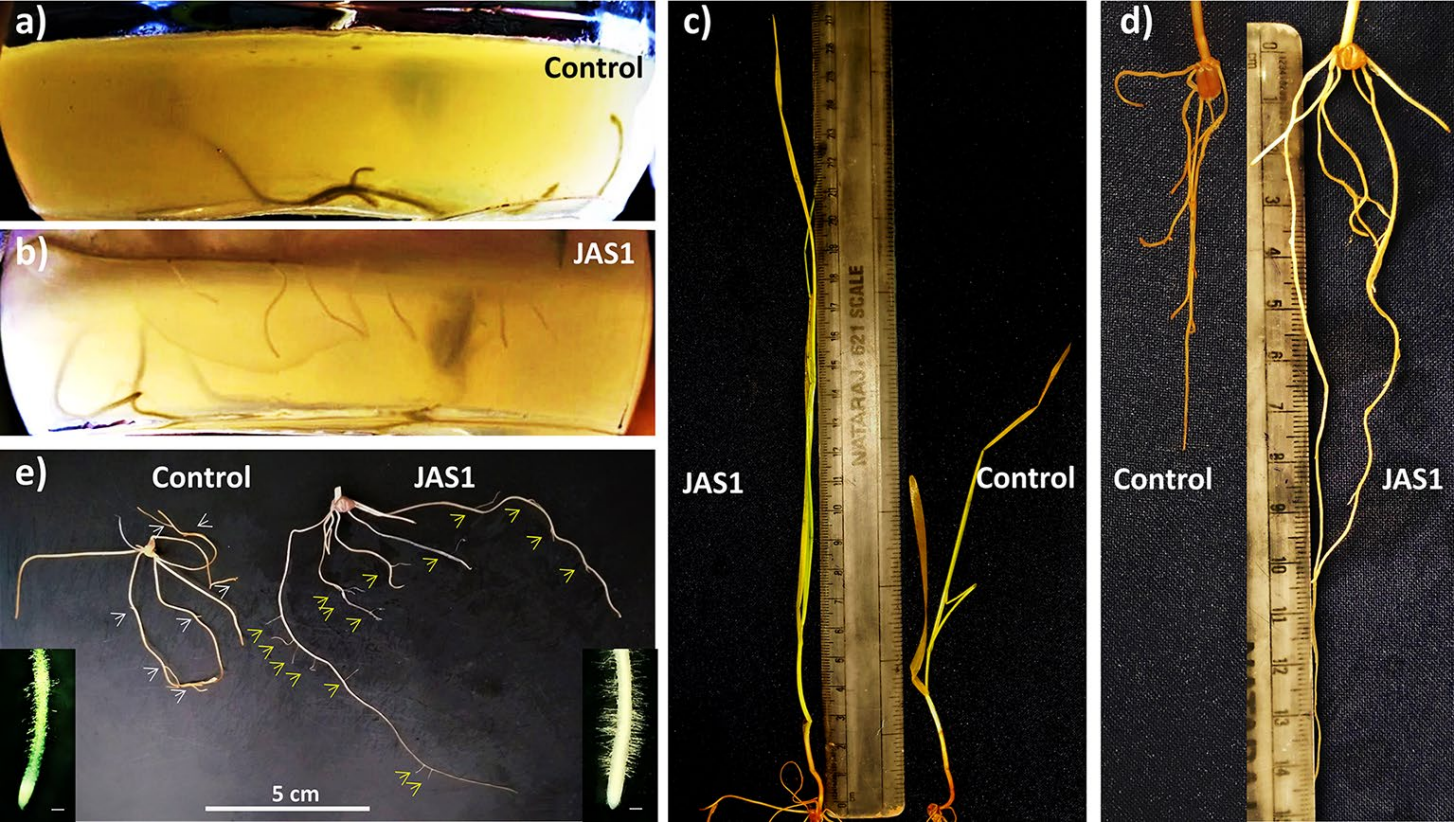
PGP effects of JAS1 over in vitro wheat seedlings. In comparison to (**a)** the uninoculated control trial, (**b)** JAS1 co-cultured with wheat seedlings shown in a jam jar (note the hazy EPS secretion over media with JAS1 overlay) increased wheat (**c)** shoot length; (**d)** root length; (**e)** root branching (indicated with yellow arrows), and root hair length and density (insets in **e** with small bars scaled to 0.5 mm). Figures representing results from three independent trials.

To correlate with the PGP effects of JAS1 in tray trials (discussed above), we also attempted it’s in vitro co-cultivation with surface sterile wheat seeds, established for a month on MSA in PTC jam jars (Fig. 9a,b). As expected, *wrt* that in controls, profound lengthening of shoots (Fig. 9c), roots (Fig. 9d), and an apparent increase in root branching (Fig. 9e) were evident. Other than these, bacterization also enhanced root hair growth both in its length and density (insets in Fig. 9e) as also seen respectively with tray trials. The root branching effect from JAS1 (in vitro), however, was prominent throughout the length on lateral (feeder) roots (Fig. 9e) as opposed to the lateral root branching points occurring immediately close to the root-shoot interface (in tray trials). These prominently branching feeder roots themselves appeared hovering over the culture media surface and rarely penetrating the media themselves except for their new branch outgrowths and otherwise also the primary roots (Fig. 9a,b,e). This may seek its support in that feeder (lateral) roots could be affinitive to EPS secreted readily by JAS1 overgrowing on the upper media profile (Fig. 9b) *wrt* control (Fig. 9a). Also, possibly branching could hail from the high IAA (biosynthesis from JAS1 as shown before) released along with the EPS. Other than these, gradually expanding EPS overlay on the media surface obviates more surface fluidity offering lesser relays, especially to the extending roots in agar (compared to that understandable with 0.8% gel strength or compactness).

In the case of tray trials, such compactness would be drastically higher in silica-contained soils, the latter which also contracts during soil drying phases showcased with the cracking phenomenon (Fig. 8b). In such scenarios, feeder roots might prefer extending more at the upper gel (and analogously also in topsoil) profile than traversing steep depths provided EPS overlaying is provisioned from JAS1. More prominent length enhancement and branching frequency in these feeders would allow for easy and quick acquisition of readily available surface water and dissolved nutrients in the substratum, especially in the topsoil profile. More branching on the surface localized feeder roots would also alternatively be considered with the substratum type and inoculum density in overlay and space limits under in vitro trials, while in ex vitro tray trials root branching on sections nearing the shoot–root interfaces or more appropriately the topsoil profile could effectuate much proportionately possibly due to locally higher IAA sequestration following the booster dosing of JAS1 hence, it may obviate higher PGP effects of the endophytes localized in this region. A better shoot growth would corroborate in light of the above effects on root and/or other PGP activities in the root, soil, and whole plant delivered by the endophyte, its EPS, and/or both^189^. Additional growth enhancement from JAS1 treatment in contrast to control sets seen on in vitro wheat seedlings propagated over a widely used plant-specific MS media composition would thus vouch for the positive PGP properties sufficed by JAS1.

### JAS1 enhances the growth of chickpea

**Figure 10:**
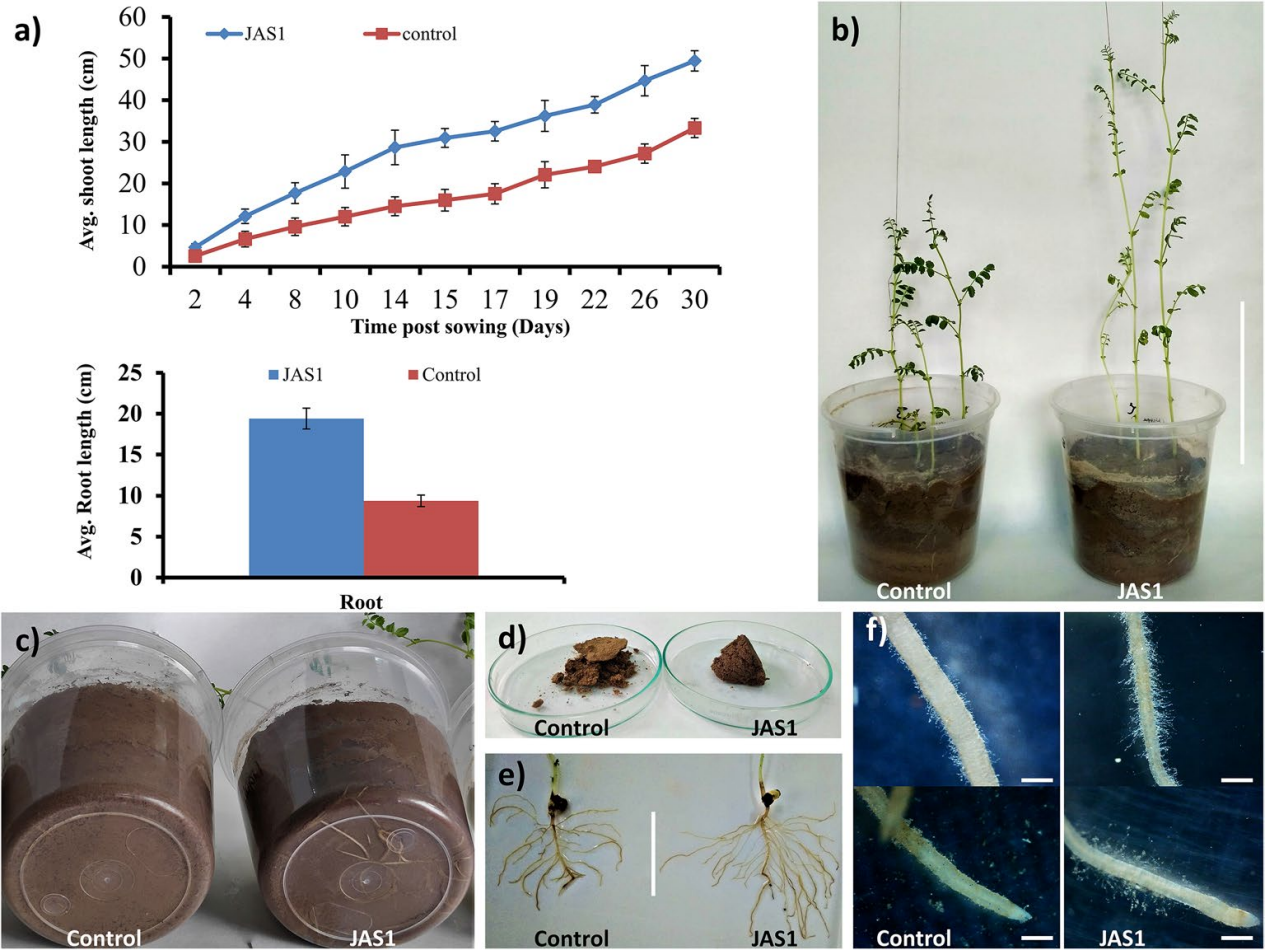
Ex vitro PGP effect of JAS1 on chickpea. **(a**) Trend with enhanced shoot development in height, bar measures 10 cm; (**b)** representative specimens with an increase in height comparative to that in control, bar measures 10 cm; (**c)** roots’ reach to the pot bottom; (**d)** scoop of soil from control and compared to JAS1 treated soil showing apparent water retention in effect from JAS1 at 30th day post sowing; (**e)** effect on secondary root development, bar measures 5 cm; and (**f)** effect on root hair density and length as seen under a stereomicroscope, bars measures 500 µm. Effects and measurements shown in (**b–f**) (except **d**) correspond to recordings made on the 15th day-day post-sowing.

Like in wheat we uncovered similar PGP effects of JAS1 upon trials also with a commercially grown legume crop, chickpea. JAS1 treatments offered significantly better growth, witnessed in the higher shoot length increments compared to plants in the control sets (Fig. 10a,b). This effect was evident right after two days from the sowing date and the trend (higher shoot length in JAS1 than in controls) remained consistent till the end of the experiment (Fig. 10a). Within about 15 days post sowing, shoots (in sets under JAS1 treatment) were seen with an averagely doubled length than those in the control treatment (Fig. 10a,b). JAS1 led growth enhancement was also evident with the roots which reached the bottom of the pot more rapidly (in almost all pots *wrt* control; Fig. 10c). Both these effects correlate with those seen with JAS1 trialed over wheat and could hence be more supportively attributed to the positive PGP effects of JAS1 (assayed biochemically before). As surmised from Fig. 10d, where a scoop was randomly taken from the top soils (from pots under trials respectively on the 30th day while the watering regime was halted beforehand on the 27th day), JAS1 treated soils remained moist even after the end of the watering regime (tightly held soil particles maintaining the scoop shape) as compared to the dried-out control soils (loosely held soil which could not maintain the scoop shape). Justifiably, JAS1 could productively synthesize EPS (as already assayed and shown before) while being attached to plants throughout the trial (as well as when applied with a booster dose) and helped the soil by retaining enough water. Observations post soil drain-out (Fig. 10e,f) suggest significant changes to root architecture in JAS1 treated chickpea *wrt* the control treatment. Comparatively, most of the roots under JAS1 treatment (except the primary) were slightly thinner (Fig. 10e,f), however, primary root length (Fig. 10e), as well as both secondary roots’ number and length, exhibited significant increments (Fig. 10e). Additionally, as seen in other plant trials (Figs. 5, 7, and 9), JAS1 treatment with chickpea also offered a substantial increase in root hair density and length (Fig. 10f, magnified root mid-sections in upper panels and sections close to and including the tip). Nodule formation, however, could not be seen in any chickpea replicates in any of the trials as opposed to that reported to effect with an IC59 strain of AP^34^.

### Plant physiological profiles under JAS1 treatment

**Figure 11:**
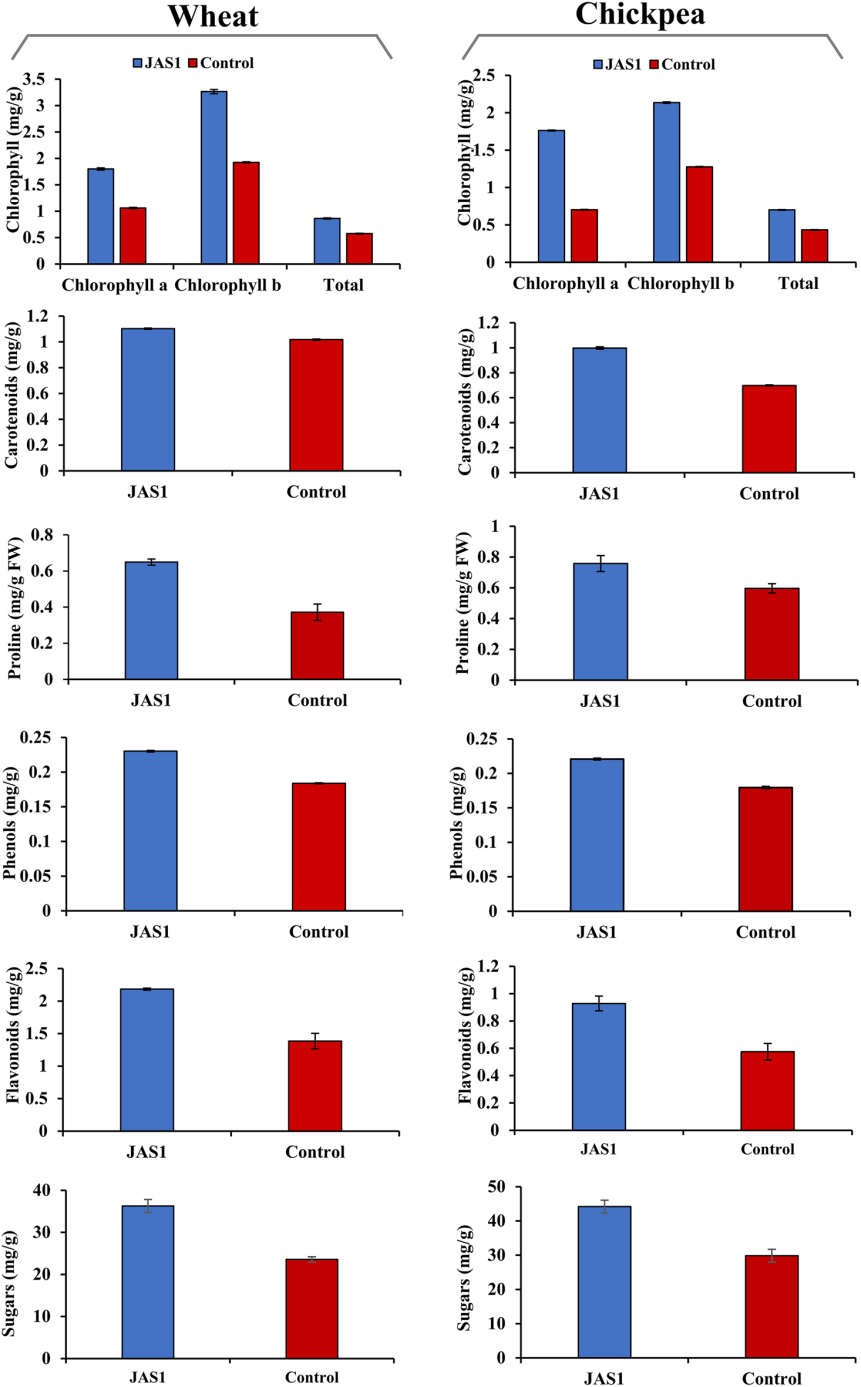
Biochemical estimates of plant growth under ex vitro treatment with JAS1. Data for both wheat and chickpea show the total available chlorophyll, carotenoids, proline, phenols, flavonoids, and soluble sugar profiles. Each dataset represents means of the means of three independent experiments each with randomly sampled 5 replicates respectively from control and JAS1 treatments. Values for phenols and flavonoids are relative to their gallic acid and quercetin equivalents, respectively.

To validate growth enhancement in both wheat and chickpea seedlings under independent treatments with JAS1, changes in the standard physiological plant growth parameters viz., total chlorophylls, carotenoids, proline, phenols, flavonoids, and sugars were analyzed (Fig. 11). Overall chlorophyll content was significantly enhanced by 50.03% and 61.71% in JAS1 treated wheat and chickpea seedlings, respectively. Chlorophyll a and b contents were also shown to increase in both wheat (69.65% and 69.56% res.) and chickpea (150.64% and 67.19% res.). Similarly, *wrt* the control treatments, JAS1 affected significantly enhanced total carotenoids in wheat (8.35%) and with a drastic increase in chickpea (42.98%). Such enhancements in the photosynthetic pigments’ profile would understandably corroborate to overall productive physiology in plants^190^. Proline is an amino acid and a key osmolyte that offers macromolecular stability to organellar structures, signal factors, enzymes, and membranes within the cells and is vital to various housekeeping tasks such as redox balancing for stress adaptations^191,192^. JAS1 treatment significantly enhanced total proline content in both wheat (74.6%), and chickpea (27.03%). Phenols protect plants under abiotic stressors by deactivating ROS and effectuating anti-oxidation^193^, and as such total phenols’ content was witnessed significantly incremented in both wheat (25.23%) and chickpea (22.98%) under JAS1 treatment as compared to controls. Nonetheless, total flavonoids as well increased in wheat (57.68%) and chickpea (61.47%) under JAS1 treatments. These include specialized secondary metabolites which accumulate to counteract various plant stresses^194^. Enhanced physiological profiles under PGPR treatments would well verse the plants’ photosynthetic performance, which should interpret an increase in total leaf sugar content^195^, as significantly accounted in both wheat (53.89%) and chickpea (48.02%) under JAS1 treatments. Sugar accumulation also fosters osmoregulatory requisites in plants, especially under stress^196^.

Taken together, these (Fig. 11) and the outcomes from various plant treatments (Figs. 5, 6, 7, 8, 9 and 10) support the assayed PGP credentials (Table 1) of the novel AP strain, JAS1, while its EPS production attribute either exclusively improves soil health by optimizing water retention, reducing desiccation-cracking (Fig. 8b,c); and/or also, understandably by virtue of the PGP activities, that contribute to soil health variously via mineralization, ammonification and other processes.

We show that JAS1 priming enhances the root branching and root hair density in ST, wheat, and chickpea. Root hairs appear as single tubular cells, commissioned for upsizing the rhizospheric area and thereby enhancing water and mineral uptake. They are the preferred sites for entry and colocalization by PGPRs, deeming particularly advantageous for plants thriving in low phosphorus soils^197–200^. Rock phosphates are insoluble and hence immobile components in soil and pose a criticality for plants by being such in a non-assimilable form of the microelement. Some plants secrete exudates to acidify soils and solubilize such phosphate forms^201^ and/or otherwise recruit their phosphate solubilizing endophytes for this^202–204^. Many of these endophytes also concomitantly ease nutrient acquisitions in plants by productive remodeling of root architecture and its development viz., growth in size and number of roots, their branching, and root hairs^202,205–208^. We glimpsed JAS1 colocalizing in root hairs of JAS1-primed PTC-raised ST explants (Supplementary Video 3) and intriguingly are exploring its transmissibility further up beyond the root-shoot interface (data not shown)**.** Nonetheless, root hair length and density may correlate positively to high auxin content in the root tip, the latter has been shown recently to stimulate Arabidopsis growing over high Cd and As soils^209^. As shown, JAS1 delivers phosphate solubilization, and high IAA content and enhances root hair length and density besides other root growth parameters (see sections with ST, wheat, and chickpea treatments with JAS1). Surprisingly, by a mechanism yet unknown, ST roots are reported to show a high absorption and low translocation of soil Cd^70^. Possibly PGP endophytes contribute to this and the above features in such plants by eliciting auxin levels^210^. Reckoning that *Sansevieria* succulents can survive well in low fertility soils and bear the dexterity to withstand various biotic and abiotic cues, presumably endophyte(s) like JAS1 and others may suffice it and other *Sansevieria* species in sustenance as resilient succulents.

## Conclusions

**Figure 12:**
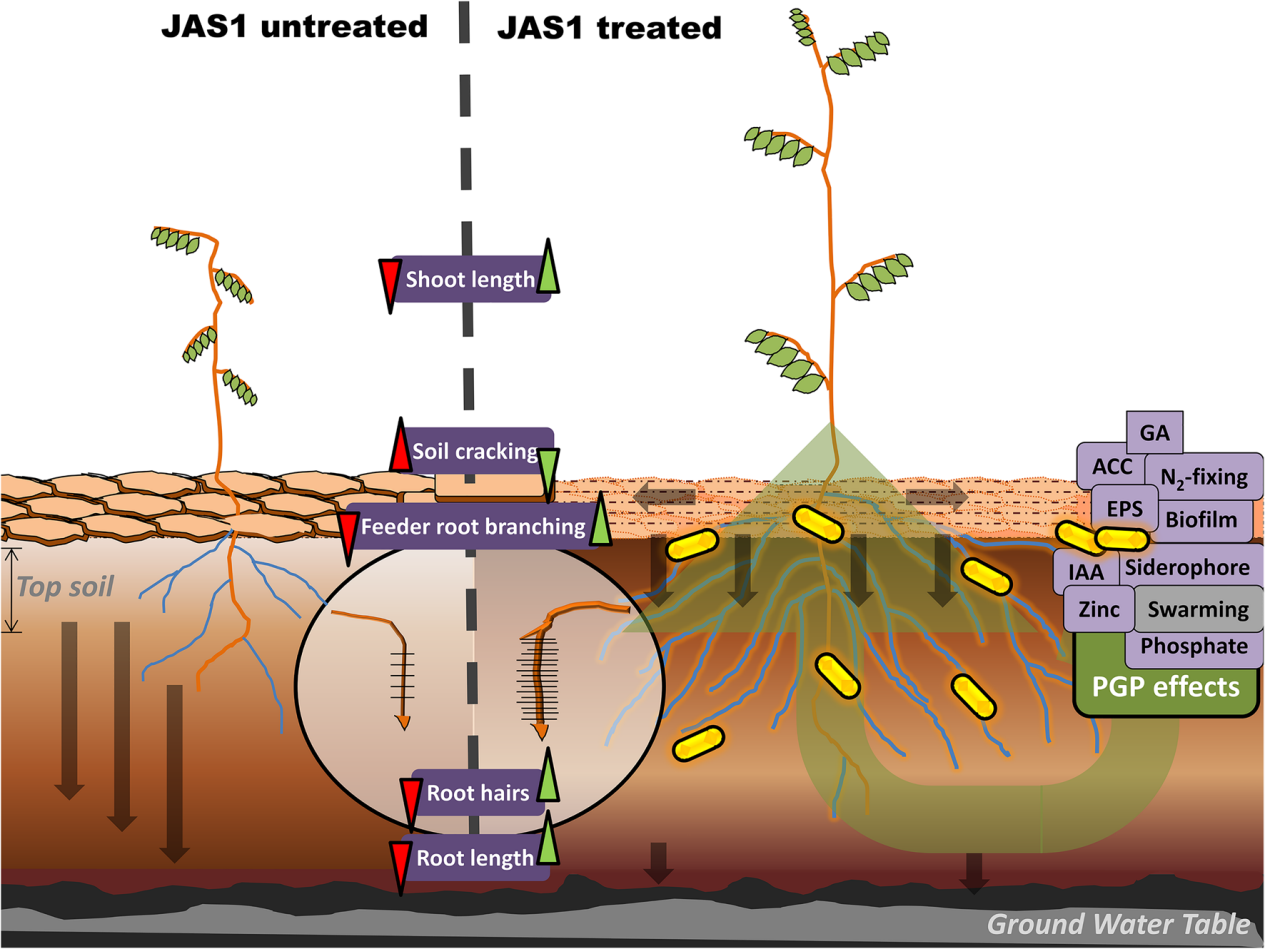
A compelling view of JAS1’s prospects: PGP properties (shown in grey boxes on the right) JAS1 (shown as yellow rods) positively influence shoot and root growth parameters (positive influences without JAS1 treatment shown with green arrow-heads, yellow for control treatment) as well as by its EPS production enhance soil water holding capacity and negates soil cracking, a usual symptom of low soil moisture which variously impacts plant performance and soil microflora. Water retention and soil holding capacity would also be greatly enhanced with a JAS1-induced increase in root branching, root hair length, and density especially in the topsoil region, thus collectively obviating enhanced and prompt absorption of minerals and growth-related materials from the soil. JAS1 and its secreted EPS may work (as other PGPRs) to enrich the soil micro and macroflora by restricting and allowing the growth of pathogenic invasive microbes and attracting other species with symbiotic interests.

This manuscript stands as the first report on the isolation of a novel AP strain, JAS1, from the genus *Sansevieria* and as the second to any reported successful attempts on isolation, characterization, and bioprospecting of any bacterial endophyte(s) from *Sansevieria* plants. The first study reported a PGP (IAA producing and ACC deaminating)-cum-TMA resistant bacterial endophyte, *Bacillus* *cereus* strain EN1, isolated from *S.* *kirkii*, which was shown to enhance the TMA removal potential of its host^87^. In our study, the JAS1 strain of AP (isolated from ST) tested positive for multiple PGP properties, besides also for enormously producing EPS. As depicted by various morphological and physiological parameters, JAS1 leads to growth enhancement in wheat and chickpea seedlings, as also apparent in its priming trials with the ST host. Besides plants’ growth requisites, water is essential for soil dexterity to enable mineralization, bio-fertility processes, and productive microbial dynamics within it. Conventional cropping systems have always faced the dilemma of low irrigated soils due to uneven rainfall, quick water seepage down to the underground table, and only a small fraction of it is available to crops and for a limited time, especially in sandy soils and/or areas under drought. Under an intermittent soil drying regime, JAS1 and its EPS could minimize desiccation-cracking by enhancing soil water retention. This presumably also rescued and rejuvenated wheat seedlings from drought stress. These multitudes of attributes in JAS1 which collectively improve both plant and soil health are summarized in a compelling infographic (Fig. 12). Bioprospecting of PGP endophytes isolated from *Sansevieria* and other succulent plants would prove a boon as newer bio-inoculants for improving commercial crop and soil performances. Other than these, heightened production of bioactives like EPS in these endophytes would allow considering cost-effective alternatives for industries relevant to its plethora of applications.

## Supplementary Information

Supplementary Information.

Supplementary Video 1.

Supplementary Video 2.

Supplementary Video 3.

## Data Availability

All data generated or analyzed during this study are included in this published article [and its supplementary information files]. All deduced nucleotide sequences in the bacterial isolate corresponding to its 16S rRNA, *recA* and *atpD* genes were deposited in NCBI’s GenBank (with publicly available accession numbers MW827601, MZ741443 and MZ741444, respectively).
