# Supplementary material for: An exopolysaccharide-producing novel Agrobacterium pusense strain JAS1 isolated from snake plant enhances plant growth and soil water retention
Source: Sci Rep. 2022 Dec 9;12:21330. doi: 10.1038/s41598-022-25225-y (PMC9734154; doi:10.1038/s41598-022-25225-y)
Supplement: Supplementary file 1 — Supplementary Information. [file 41598_2022_25225_MOESM1_ESM.docx]

**Supplementary resources**

**Table S1. Biochemical, morphological characterizations, and enzyme activity assays.**

| ***Assay classes*** | ***Activity/assays*** | | ***Characteristics*** | | | |
| --- | --- | --- | --- | --- | --- | --- |
| **Morphology and growth responses** | | |  | | | |
|  | Gram reaction | -ve | |  |  |  |
|  | Shape | Rod-shaped | |  |  |  |
|  | Colony on NA | Smooth, irregularly shaped, pale white, swarming motility | |  |  |  |
|  | Colony on MSA | Smooth, white, mucoid, oozy, with a highly viscous bulge | |  |  |  |
|  | Colony on LBA | Smooth, irregularly shaped, white, swarming motility | |  |  |  |
| **Standard biochemical responses** | | | | | |  |
|  | Catalase test | | + | | |  |
|  | Methyl red | | + | | |  |
|  | Indole test | | + | | |  |
|  | Citrate utilization | | - | | |  |
|  | Voges Proskauer | | - | | |  |
|  | Starch hydrolysis | | - | | |  |
|  | Urease test | | - | | |  |
|  | Oxidase test | | - | | |  |
|  | Nitrate reduction | | - | | |  |
|  | Motility test | | + | | |  |
|  | Hydrogen sulfide test | | - | | |  |
|  | Tween-20 hydrolysis | | - | | |  |
|  | Tween-80 hydrolysis | | - | | |  |
|  | α-ketolactose utilization | | - | | |  |
| ***Carbohydrate utilization*** | | | | | |  |
|  | Glucose | | + | | |  |
|  | Sucrose | | + | | |  |
|  | Starch | | + | | |  |
|  | Mannitol | | + | | |  |
|  | Lactose | | - | | |  |
|  | Dextrose | | + | | |  |
|  | Fructose | | + | | |  |
|  | Gelatin | | - | | |  |
|  | Arabinose | | - | | |  |
|  | Adonitol | | - | | |  |
|  | Sorbitol | | - | | |  |
|  | Rhamnose | | - | | |  |
| ***Growth in NaCl*** | | | | | |  |
|  | 0% | | + | | |  |
|  | 1% | | + | | |  |
|  | 2% | | - | | |  |
|  | 3% | | - | | |  |
|  | 4% | | - | | |  |
|  | 5% | | - | | |  |
| ***Enzyme activities*** | | |  | | |  |
|  | Cellulase | | - | |  |  |
|  | Protease | | + | |  |  |
|  | Lipase | | - | |  |  |
|  | Pectinase | | - | |  |  |
|  | Amylase | | - | |  |  |

**Table S2. Antibiotic sensitivity of JAS1 isolate.** Disc size= 6mm; S=Less susceptibility (7-10mm); S^+^=Susceptibility (11-20mm); S^++^= High susceptibility (21-30mm); S^+++^= Extreme susceptibility (31-40mm); R=Resistant (0 mm). All antibiotics were purchased from Himedia with indicated catalog number in the first column.

| ***Cat#*** | ***Antibiotic (Concentration)*** | **JAS1** | |
| --- | --- | --- | --- |
|  |  | ***Inhibition zone (mm)*** | ***Response*** |
| SD039 | Trimethoprim (5μg) | 0 | R |
| SD731 | Neomycin (10μg) | 23±0.81 | S^++^ |
| SD031 | Streptomycin (10μg) | 8.66±0.47 | S |
| SD181 | Spectinomycin (10μg) | 22.33±0.47 | S^++^ |
| SD040 | Cefotaxime (30μg) | 19.33±0.47 | S^+^ |
| SD019 | Methicillin (5μg) | 0 | R |
| SD028 | Penicillin G (10 units) | 0 | R |
| SD192 | Clarithromycin (15μg) | 21.33±0.47 | S^++^ |
| SD133 | Tetracycline (10μg) | 30.66±0.47 | S^+++^ |
| SD006 | Chloramphenicol (30μg) | 12.33±0.47 | S^+^ |
| SD060 | Ciprofloxacin (5μg) | 37±0 | S^+++^ |
| SD061 | Cefuroxime (30 μg) | 0 | R |
| SD016 | Gentamicin (10 μg) | 23.33±1.24 | S^++^ |
| SD002 | Ampicillin (10 μg) | 0 | R |
| SD045 | Vancomycin (30μg) | 0 | R |
| SD065 | Ceftriaxone (30 μg) | 0 | R |
| SD035 | Amikacin (30 μg) | 16.16±0.62 | S^+^ |
| SD050 | Cephalothin (30 μg) | 10.83±0.23 | S^+^ |
| SD010 | Co-Trimoxazole (25 μg) | 0 | R |
| SD073 | Imipenem (10 μg) | 18.66±0.47 | S^+^ |
| SD727 | Meropenem (10 μg) | 26.66±0.94 | S^++^ |
| SD044 | Tobramycin (10 μg) | 12.33±0.47 | S^+^ |
| SD217 | Moxifloxacin (5 μg) | 25.66±0.47 | S^++^ |
| SD087 | Ofloxacin (5 μg) | 27.33±0.47 | S^++^ |
| SD162 | Sparfloxacin (5 μg) | 18.33±1.24 | S^+^ |
| SD216 | Levofloxacin (5 μg) | 28±0.81 | S^++^ |
| SD001 | Amoxicillin (10 μg) | 14.83±0.23 | S^+^ |
| SD075 | Cloxacillin (5 μg) | 0 | R |
| SD013 | Erythromycin (15 μg) | 16.33±0.94 | S^+^ |
| OD026R | Penicillin V (30 μg) | 8.66±0.47 | S |
| SD048 | Cephalexin (30 μg) | 21.83±0.23 | S^++^ |
| SD017 | Kanamycin (50 μg) | 27.16±0.23 | S^++^ |
| SD030 | Rifampicin (50 μg) | 20.5±0.40 | S^++^ |
| SD021 | Nalidixic acid (50 μg) | 33.5±0.70 | S^+++^ |
| SD051 | Clindamycin (2 μg) | 0 | R |
| SD184 | Norfloxacin (5 μg) | 32.33±0.94 | S^+++^ |

**Legends to Supplementary Videos:**

**Video 1. Close view of a JAS1 swarm front.** The first 8 seconds of the video-clipping show a multi-layered swarm front of JAS1 developed on nutrient agar. Freely moving bacteria can be seen at the edges (site marked by an arrow), probably in process of registering a new spiral formation in the swarm front. In the next 8 seconds, the marked site is shown magnified to vividly document free movements.

**Video 2. Swimming motility of JAS1 populations dislodged from swarms.** A 4-second clipping depicts the free-swimming activity of a bacterial population that got disembarked from their swarm. The swarm front on nutrient agar was disturbed using a fine aseptic needle.

**Video 3.** **JAS1 colocalizing in the root hairs of PTC raised ST.** The video depicts that JAS1 could easily penetrate and colocalize within the root hairs.

**Media and reagents**

| **CMC agar** | Nutrient agar supplemented with 1% carboxymethylcellulose; pH 6.8 |
| --- | --- |
| **PSAM** | 3 g diammonium hydrogen phosphate, 2 g monopotassium phosphate, 3 g dipotassium phosphate, 0.1 g magnesium phosphate, 15 g agar, 10 g pectin for 1 liter; pH 4.5 |
| **SAM** | 10 g soluble starch, 0.5 g potassium nitrate, 1 g dipotassium hydrogen phosphate, 0.2 g magnesium sulfate heptahydrate, 0.1 g calcium chloride, traces of ferric chloride, 15 g agar for 1 liter; pH 7.0 |
| **NBRIP medium** | 10 g glucose, 5 g tricalcium phosphate, 5 g magnesium chloride hexahydrate, 0.25 g magnesium sulphate heptahydrate, 0.2 g potassium chloride, 0.1 g ammonium sulphate, and 0.025 g bromophenol blue for 1 liter; pH 7 |
| **Molybdate reagent** | 0.42 M sulphuric acid; 0.2% ammonium molybdate; 0.175% ascorbic acid; 0.005% potassium antimony |
| **DF minimal salt medium** | 4 g KH_2_P0_4_, 6g Na_2_HPO_4_, 0.1 g FeSO_4_.7H_2_O, 10 µg H_3_BO_3_, 0.2 g MgSO_4_.7H_2_O, 70µg ZnSO_4_, 50 µg CuSO_4_, 10 µg MnSO_4_, 2 g Glucose, 2 g gluconic acid, 10 µg MoO_3_, 2 g citric acid, 12 g agar, for 1 liter |
| **CAS reagent** | Preprare with sequential mixing of the following three solutions, i) 100 ml of 2 mM Chrome Azurol S; ii) 20 ml solution of 1 mM FeCl_3_ prepared in 10 mM HCl; and iii) 20 ml of 5 mM HDTMA (hexadecyl trimethyl ammonium bromide) |
| **Semisolid basal media** | 1% glucose, 0.1% ammonium sulphate, 0.02% potassium chloride; 0.01% dipotassium hydrogen phosphate, 0.02% magnesium sulphate, 1.5% agar, pH 7.0 |
| **Aleksandrow agar media** | 1% C₆H₁₂O₆, 0.5 g MgSO_4_, 0.005 g FeCl_3_, 0.1 g CaCO_3_, 2 g Ca_3_(PO_4_)_2_, 5 g AlKO_6_Si_2_, 3% agar; pH 6.5 |
| **Jensen media** | 2% sucrose, 1.0 g K_2_HPO_4_, 0.5 g MgSO_4_, 0.5 g NaCl, 0.005 g Na_2_MoO_4_, 0.01 g FeSO_4_, 2 g CaCO_3_, 1.5% agar, pH 7, make up to 1 liter |
| **Congo Red Agar (CRA)** | mix 0.8 g Congo red, 50 g sucrose with 37 g brain heart infusion media (Himedia, Mumbai, India) in a liter preparation gelled with 15 g agar; pH 7.2 |
